# KLF1 coordinates specialized transcriptional networks required to maintain the integrity of terminal erythropoiesis

**DOI:** 10.1242/jcs.264036

**Published:** 2025-11-13

**Authors:** Merlin N. Gnanapragasam, Peng Jiang, Anita R. Dhara, Parina N. Patel, Mahesh Ramamoorthy, Roberta B. Nowak, Velia M. Fowler, James J. Bieker

**Affiliations:** ^1^ Department of Cell, Developmental, and Regenerative Biology, Mount Sinai School of Medicine, New York, NY 10029, USA; ^2^ Department of Biological, Geological, and Environmental Sciences, Cleveland State University, Cleveland, OH 44115, USA; ^3^ Center for Gene Regulation in Health and Disease, Cleveland State University, Cleveland, OH 44115, USA; ^4^ Department of Molecular Medicine, The Scripps Research Institute, La Jolla, CA 92037, USA; ^5^ Division of Signaling and Gene Expression, La Jolla Institute for Immunology, La Jolla, CA 92037, USA; ^6^ Department of Biological Sciences, University of Delaware, Newark, DE 19716, USA

**Keywords:** Transcription, Enucleation, Cytokinesis, Erythropoiesis

## Abstract

Krüppel-like factor 1 [KLF1; also known as erythroid Krüppel-like factor (EKLF)] is a C2H2 zinc finger transcription factor that plays a crucial role in all aspects of erythropoiesis. Mutations in KLF1 lead to diverse phenotypes ranging from mild to severe anemias. Individuals with a heterozygous E325K mutation [congenital dyserythropoietic anemia (CDA) type IV] exhibit impaired erythroid terminal differentiation and increased presence of binucleate erythroblasts. We have previously shown that KLF1 is necessary for cell cycle exit and enucleation in mouse primary cells. In the present study, we discovered that genes involved in cell motility, cell division and mitotic pathways are all directly regulated by KLF1. *Klf1*^−/−^ cells exhibit increased numbers of binucleated erythroblasts and DNA bridges, and differentiating *Klf1*^−/−^ erythroblasts display an increased percentage of cytokinesis failure events and defective microtubule bundling. *Klf1*^−/−^ erythroblasts produce frequent aberrant F-actin-rich membrane protrusions and anucleate cell fragments. Human CDA type IV cells exhibit similar patterns of dysregulation of cytokinesis and cell motility genes. Collectively, we show that KLF1 is necessary for maintaining the integrity of erythroid cell divisions by direct regulation of genes involved in cytokinesis and motility pathways during terminal erythroid differentiation.

## Teaser

**Summary:** The transcription factor Krüppel-like factor 1 (KLF1), which plays a crucial role in erythropoiesis, also plays a role in cell motility, cell division and mitotic pathways necessary for the integrity of terminal erythroid differentiation.

## INTRODUCTION

Krüppel-like factor 1 [KLF1; also known as erythroid Krüppel-like factor (EKLF)] is the founding member of the Krüppel-like family of C2H2 zinc finger transcription factors (Miller and Bieker, 1993). KLF1 is a master regulator of erythropoiesis and has an erythroid-enriched expression (Miller and Bieker, 1993; Nuez et al., 1995; Perkins et al., 1995). It binds to the DNA consensus sequence 5′-CCMCRCCCN-3′ (M, amino nucleotide; R, purine nucleotide; N, any base) at its target genes via three zinc fingers at its carboxyl end (Miller and Bieker, 1993). It orchestrates the expression of a wide range of targets that are crucial for erythropoiesis, including having a direct role in controlling the developmental switch of hemoglobin gene expression (Bieker and Philipsen, 2024; Perkins et al., 2016; Siatecka and Bieker, 2011).

Mammalian terminal erythroid development is a precisely regulated process involving rapid terminal cell divisions and series of well-orchestrated changes to form mature erythrocytes (Lodish et al., 2010; Newton et al., 2024). This process involves the commitment of erythroid progenitors to give rise to proerythroblasts, basophilic erythroblasts, polychromatic erythroblasts and orthochromatic erythroblasts, which enucleate to form reticulocytes that further mature in circulation into red blood cells (Lodish et al., 2010; Shearstone et al., 2010). Erythroid differentiation is marked by a gradual decrease in cell and nuclear size, and an increase in nuclear condensation and polarization. Cell surface markers such as Ter119, CD44 and CD71, along with cell size parameters such as forward scatter, are used to identify erythroid cells at various stages of terminal erythroid differentiation (Chen et al., 2009; Shearstone et al., 2010). However, many of these markers are dysregulated in their expression in *Klf1*^−/−^ erythroid cells (Gnanapragasam et al., 2016), leading to challenges in delineating the functions of KLF1 at late differentiation stages.

In order to study the functions of KLF1 during terminal erythroid differentiation without relying on cell surface markers, we previously utilized imaging flow cytometry to characterize and define cells at various stages of erythropoiesis, by measuring cell size, nuclear size and nuclear polarization parameters (Gnanapragasam et al., 2016). In addition, we employed a murine *ex vivo* erythroid culture system that enriches for extensively self-renewing erythroblasts (ESREs), which can be induced to terminally differentiate and enucleate in 3 days (England et al., 2011). We characterized these erythroid cultures derived from *Klf1*^+/+^ and *Klf1*^−/−^ embryonic day (E)12.5 fetal livers (Gnanapragasam et al., 2016), given that *Klf1*^−/−^ is embryonic lethal by E14.5 (Nuez et al., 1995; Perkins et al., 1995). These studies showed that the absence of KLF1 causes a two-stage block, leading to an accumulation of early progenitors due to a premature cell cycle exit (Pilon et al., 2008), and a later stall in differentiation at the orthochromatic erythroblast stage accompanied by a complete block in enucleation and impaired cell cycle exit (Gnanapragasam and Bieker, 2017; Gnanapragasam et al., 2016).

KLF1 variants in humans lead to diverse phenotypes ranging from a benign increase in fetal hemoglobin levels to severe anemia (Bieker and Philipsen, 2024; Perkins et al., 2016; Waye and Eng, 2015). One such pathogenic variant that we and others have characterized is a heterozygous missense change in the zinc finger domain of KLF1 (p.E325K) that leads to congenital dyserythropoietic anemia (CDA) type IV (Arnaud et al., 2010; Jaffray et al., 2013; Singleton et al., 2011). This variant results in ineffective erythropoiesis and an increased presence of binucleate erythroid cells in the bone marrow (OMIM 613673). A high prevalence of bi- and multi-nucleated cells is a characteristic feature of this group of congenital dyserythropoietic anemias (CDAs). The aberrant character of CDAs arises due to severe molecular defects in DNA replication and cytokinesis pathways, which lead to impaired erythroid terminal cell divisions and the distinctive pathological phenotype of CDAs (Gambale et al., 2016; Iolascon et al., 2013). For example, variants in KIF23, a protein important for cytokinesis, can lead to CDA type III, which results in defects in erythroid precursors characterized by binucleated erythroblasts (Gambale et al., 2016; Iolascon et al., 2013; Liljeholm et al., 2013). A perplexing aspect of these disorders is how pathogenic variants in genes that play ubiquitous roles in DNA replication and cytokinesis affect the erythroid lineage selectively whereas other lineages are largely spared.

In the present study, we employ the previously characterized murine *Klf1*^+/+^ and *Klf1*^−/−^ ESRE *ex vivo* culture system (Gnanapragasam et al., 2016) to study the functions of KLF1 during terminal erythroid differentiation using genomics and functional investigations. We show that KLF1 directly regulates cell division and cell motility pathways, specifically during terminal erythroid differentiation, and that this correlates with cytokinesis defects and aberrant membrane protrusions observed in *Klf1*^−/−^ erythroid cells at late stages. In addition, a bioinformatics comparison of RNA-seq data from murine erythroid cells with data from human erythroid cells from an individual with CDA type IV (Varricchio et al., 2019), highlights their common features in the dysregulation of genes involved in cell division pathways.

## RESULTS

### KLF1 has a higher transcriptional impact on erythroid cells during terminal differentiation

Erythroid cells were obtained from *Klf1*^+/+^ and *Klf1*^−/−^ E12.5 fetal mouse C57BL6 livers for *ex vivo* cultures, given that KLF1 loss leads to embryonic lethality by E14.5. The ESREs from these cultures can be induced to undergo erythroid terminal differentiation to enrich for terminally differentiating cells without relying on cell surface markers, which are aberrantly expressed in *Klf1*^−/−^ erythroid cells. By day 3 of differentiation, *Klf1*^+/+^ but not *Klf1*^−/−^ orthochromatic erythroblasts undergo enucleation (England et al., 2011; Gnanapragasam et al., 2016). Using this system, we previously characterized defects in enucleation and cell cycle exit at the orthochromatic erythroblast stage in *Klf1*^−/−^ cells during terminal erythroid differentiation, and showed that these phenotypes were conserved *in vivo* (Gnanapragasam et al., 2016). Therefore, to globally delineate the functions of KLF1 during terminal erythroid differentiation, we harvested erythroid cells cultured in expansion medium and in erythroid differentiation medium at day 2 of terminal differentiation, similar to what was undertaken in our previous studies (Gnanapragasam et al., 2016), and performed RNA sequencing in *Klf1*^+/+^ and *Klf1*^−/−^ cells to identify genes and pathways regulated by KLF1 before and after terminal differentiation. As more fully described in the Materials and Methods, we used transcripts per million (TPM) for our present analysis. Changes based on TPM reflect relative abundance within the sequenced libraries, not absolute transcriptional activity per cell.

**Fig. 1.**
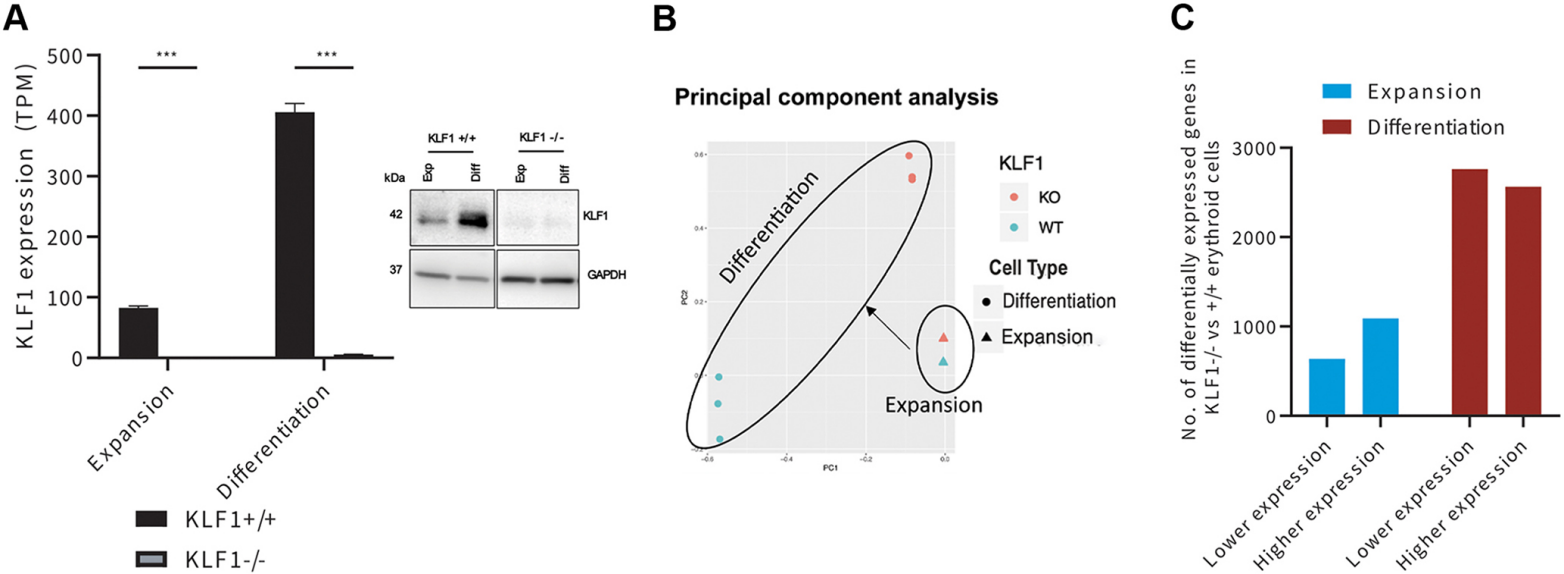
**KLF1 has a higher impact on erythroid cell gene expression during erythroid terminal differentiation than during erythroid expansion.** (A) KLF1 expression before and after differentiation at the RNA (left) and protein (right) level, comparing WT and KO cell sources. RNA from biological triplicates were analyzed for each condition. Results are mean±s.e.m. ***P<0.001 for the RNA analysis (two-tailed, unpaired *t*-test). Blot shown is representative of two repeats. GAPDH used as a control for protein loading. (B) Principal component analyses (PCA) of differentially expressed genes (DEGs) are shown for expansion and differentiation phases in *Klf1*^−/−^ (KO) and +/+ (WT) cells. Three biological replicates for each condition were analyzed. Replicates for the expansion samples appear indistinguishable as they directly overlap each other (Table S2). (C) The number of DEGs between *Klf1*^−/−^ and *Klf1*^+/+^ is greater in terminally differentiating cells than in expanding erythroid cells. Within these sets, a comparison of the number of genes expressed at a higher or lower level in the absence of KLF1 compared to *Klf1*^+/+^ erythroblasts is shown for both expansion and terminal differentiation phases. DEGs are calculated by considering all replicates together within a condition and determining the number of conditions that are significantly different (FDR<5% based on EBSeq R package and greater than 2-fold changes of average of replicates for each condition); hence, DEG is an absolute number with no error bars.

Using this method of analysis, *Klf1*^−/−^ cells showed a total absence of KLF1 mRNA and protein expression (Fig. 1A). Principal component analysis (PCA) and hierarchical analysis showed that the triplicate RNA samples preferentially clustered with each other (Fig. 1B; Fig. S1A). The ability to cleanly separate expansion from differentiation phases provides a powerful tool to assess, for the first time, the role of KLF1 at each of these erythroid developmental time points.

Our analysis revealed that a greater number of genes were differentially expressed between *Klf1*^+/+^ (WT) and *Klf1*^−/−^ (KO) cells during terminal differentiation compared to what was seen in the expansion stage (Fig. 1C). In addition, these data showed that there were more genes that were downregulated compared to upregulated during terminal differentiation in *Klf1*^−/−^ cells compared to in *Klf1*^+/+^ cells. This was not observed for housekeeping gene expression (Fig. S1B). These results show that the dysregulation of the transcriptome is exacerbated in *Klf1*^−/−^ cells during terminal differentiation.

**Fig. 2.**
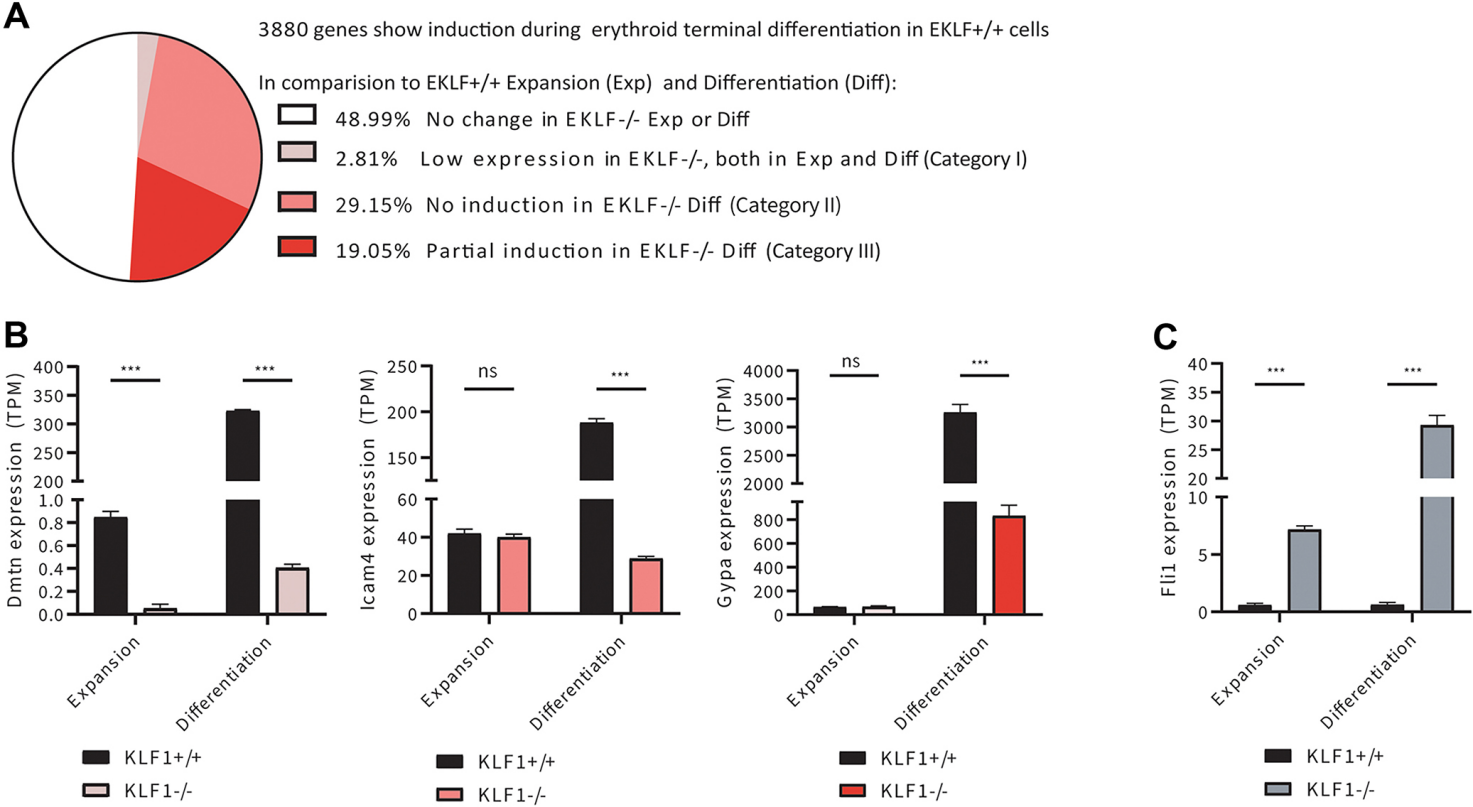
**Categories of KLF1-regulated erythroid targets.** (A) 3880 genes show induction during erythroid terminal differentiation when comparing expansion and differentiation in *Klf1*^+/+^ cells. Among the 50% of the genes that show differential expression in *Klf1*^−/−^ erythroid cells, we identified three categories: Category I (little to no expression in *Klf1*^−/−^ erythroid cells compared to *Klf1*^+/+^ erythroid cells); Category II (expression but no induction in differentiating *Klf1*^−/−^ cells compared to *Klf1*^+/+^ cells); and Category III (expression but partial induction in differentiating *Klf1*^−/−^ cells compared to *Klf1*^+/+^ cells). (B) The normalized, relative gene expression profile [transcripts per million (TPM)] for representative genes from the three categories are shown [dematin (*Dmtn*) for Category I; *Icam4* for Category II; and glycophorin A (*Gypa*) for Category III]. (C) Gene expression of *Fli1*, a megakaryocyte-specific gene that is normally repressed in erythroid cells, shows increased expression in both expanding and differentiating *Klf1*^−/−^ cells compared to *Klf1*^+/+^ cells. Data is mean±s.e.m. from biological triplicates analyzed for each condition. ***P<0.001; ns, not significant (two-tailed, unpaired *t*-test).

A closer analysis shows that among the genes that are normally induced during terminal erythroid differentiation, KLF1-regulated erythroid targets fell into three categories (Fig. 2A). One set consisted of genes that are only expressed at insignificant levels in the absence of KLF1, whether it be during expansion or differentiation (e.g. *Dmtn*; Fig. 2B). A second was genes that are equivalently expressed at a minimal level during expansion, but whose increase after differentiation does not occur without KLF1 (e.g. *Icam4*; Fig. 2B). There are numerous examples of this category that likely account for the dramatic PCA cluster differences seen after differentiation. Finally, a third group were the genes that were induced after differentiation but never attained optimal levels in the absence of KLF1 (e.g. *Gypa*; Fig. 2B). These data enable, for the first time, the means to distinguish transcription factor effects at expansion versus differentiation phases rather than a mixed assessment within the bulk fetal liver erythroid cell population.

KLF1 plays an important role in the bipotential decisions emanating from the megakaryocyte/erythroid progenitor (MEP) cell, particularly by repressing megakaryopoiesis while stimulating erythropoiesis (Bouilloux et al., 2008; Frontelo et al., 2007). Absence of KLF1 leads to ‘lineage infidelity’ whereby the nominally erythroid cell mis-expresses some of the normally repressed megakaryocyte-specific genes (Isern et al., 2010; Tallack and Perkins, 2010). KLF1–Fli1 cross-antagonism is thought to play a crucial role in this process (Frontelo et al., 2007; Starck et al., 2003). Inspection of the ESRE data supports this dramatic expression change, with *Fli1*, *Itga2b* (CD41) and *Itgb3* (CD61), not normally expressed in the KLF1 WT red cell, attaining high expression levels in the absence of KLF1 (Fig. 2C; Fig. S1C).

### Cell division and mitosis are among the top enriched pathways that are directly regulated by KLF1 specifically during terminal differentiation

**Fig. 3.**
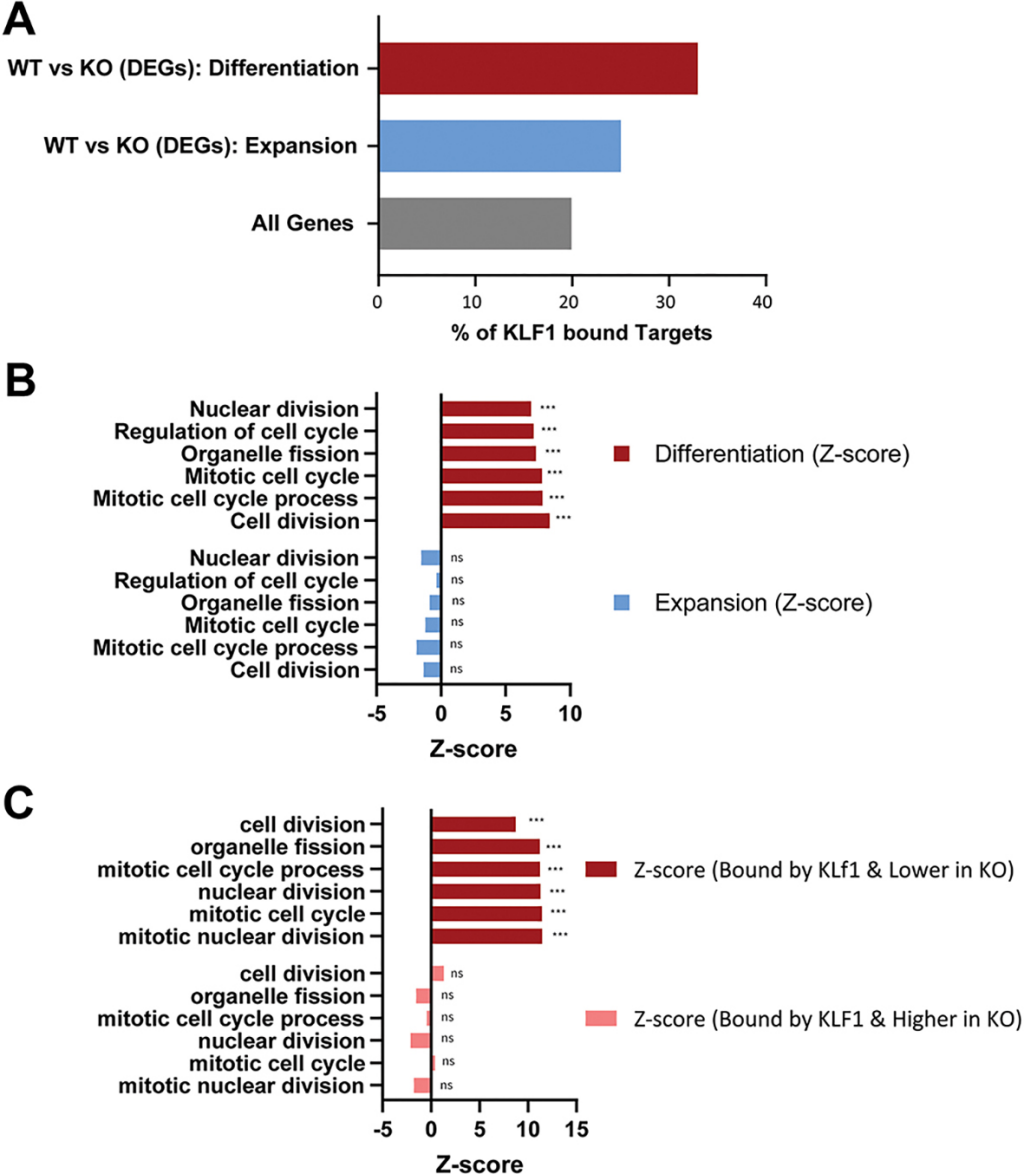
**Cell division and mitosis are among the top enriched pathways specifically for genes upregulated by KLF1 during terminal differentiation.** (A) A higher proportion of differentially expressed genes (DEGs) are bound by KLF1 in erythroid cells during terminal differentiation than during expansion. The percentage of KLF1-bound gene targets is greater among DEGs in differentiating cells than among DEGs in expanding erythroid cells, compared to baseline levels across all genes. DEGs are calculated by considering all replicates together within a condition and determining the number of conditions that are significantly different (FDR<5% based on EBSeq R package and greater than 2-fold changes of average of replicates for each condition); hence, DEG is an absolute number with no error bars. (B) Top six enriched Gene Ontology (GO) terms for differentially expressed genes between *Klf1*^+/+^ and *Klf1*^−/−^ erythroid cells during expansion and terminal differentiation and their corresponding *Z*-scores are shown. These terms are related to cell division and mitosis. The *Z*-score shows that cell division and mitosis pathways are positively enriched among genes that are differentially expressed during differentiation (red) but not during expansion (blue). ***P<0.001; ns, not significant (two-tailed, unpaired *t*-test). (C) Merging of the RNA-Seq data with previous ChIP-Seq data (Ilsley et al., 2019), shows that the cell division and mitosis pathways are directly regulated by KLF1 during differentiation and only in the genes that are upregulated by KLF1 (i.e. down in *Klf1*^−/−^) during terminal differentiation. The *Z*-score shows that the cell division and mitosis pathways are positively enriched among genes that are bound by KLF1 and more highly expressed in *Klf^+/+^* erythroid cells (i.e. lower in *Klf1*^−/−^), shown with red bars, but not among genes that are bound by KLF1 but less expressed in *Klf1*^+/+^ erythroid cells (i.e. higher in *Klf1*^−/−^), shown with pink bars. ***P<0.001; ns not significant (two-tailed, unpaired *t*-test).

In order to identify direct transcriptional targets of KLF1, we merged our RNA-Seq data with previously published KLF1 ChIP-Seq data (Ilsley et al., 2019; GEO accession GSE92620). This analysis revealed an elevated enrichment of KLF1-bound targets during differentiation and expansion compared to those at baseline levels across all genes (Fig. 3A). Notably, enrichment of KLF1-bound target genes was the highest among the differentially expressed genes during terminal differentiation compared to what is seen in expansion (Fig. 1B). These studies overall show that KLF1 binds to and transcriptionally regulates a higher number of gene targets that are expressed during terminal differentiation compared to in the expansion phase, highlighting the importance of the transcriptional activity of KLF1 for the progression of terminal differentiation.

To identify and compare the biological pathways that are regulated by KLF1 during expansion and terminal differentiation, we performed gene ontology (GO) pathway analysis using the differentially expressed gene list between *Klf1*^+/+^ and *Klf1*^−/−^ cells. Cell division and mitosis pathway terms are enriched in the top 15 GO terms, specifically during terminal differentiation (Fig. 3B). The top 20 differentially regulated genes associated with cell division based on an odds ratio are shown in Fig. S2. Next, we examined the pathways enriched among genes that are direct targets of KLF1; that is, bound by KLF1 (Ilsley et al., 2019) and differentially regulated in *Klf1*^−/−^ erythroblasts. Notably, we find that only genes upregulated by KLF1 (i.e. down in *Klf1*^−/−^) and bound by KLF1 in *Klf1*^+/+^ cells are enriched for cell division and mitosis related pathways (Fig. 3C). Overall, these analyses show that during erythroid terminal differentiation, KLF1 binds and directly upregulates genes involved in cell division pathways.

**Fig. 4.**
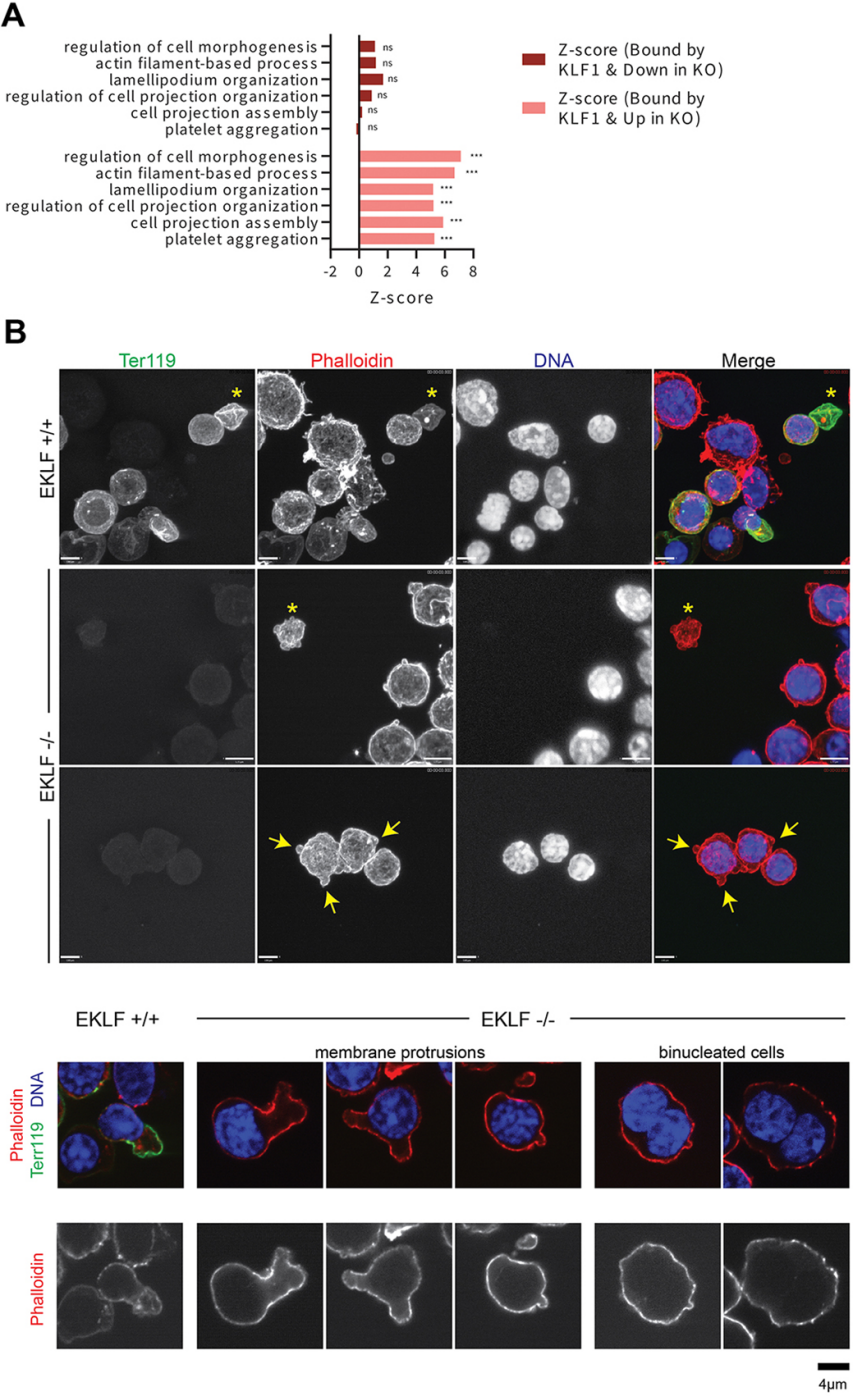
**Klf1**^−/−^ **cells are enriched in binucleate cells with aberrant motility, based on gene expression and immunostaining analyses.** (A) Merging of the RNA-Seq data with previous ChIP-Seq data (Ilsley et al., 2019), shows that pathways related to megakaryopoiesis are directly repressed by KLF1 during differentiation and only in the genes that are downregulated by KLF1 (i.e. up in *Klf1*^−/−^) during terminal differentiation. The *Z*-score shows that these pathways are positively enriched among genes that are bound by KLF1 and more repressed in *Klf1*^+/+^ erythroid cells (i.e. higher in *Klf1*^−/−^), shown with pink bars, but not among genes that are bound by KLF1 and are expressed higher in *Klf1*^+/+^ erythroid cells (i.e. lower in *Klf1*^−/−^), shown with red bars. ***P<0.001; ns not significant (two-tailed, unpaired *t*-test). (B) Immunofluorescence confocal microscopy of freshly isolated fetal liver erythroid cells (E12.5) reveals that *Klf1*^−/−^ erythroid cells are Ter-119 negative, with F-actin-rich protrusions (arrows) and anucleate cell fragments (asterisks) (top and bottom panels). Binucleate cells are also evident (bottom panel). Cells were stained with the red cell membrane marker Ter-119 (green), Rhodamine–phalloidin for F-actin at the cell cortex (red) and Hoechst 33258 for the nucleus (blue). Top panel, extended focus projections of confocal *Z*-stacks. Scale bars: 3.9 µm (top and bottom rows); 5.7 µm (middle row). Bottom panel, confocal single optical sections. Scale bars: 4 µm. Images shown are representative of three repeats.

Furthermore, consistent with previously described roles for KLF1 in the bipotential lineage decisions in MEP cells, the top dysregulated pathways among genes repressed by KLF1 (i.e. upregulated in *Klf1*^−/−^) were related to megakaryocytic differentiation, such as actin filament organization, lamellipodium organization and platelet aggregation (Bouilloux et al., 2008; Frontelo et al., 2007) (Fig. 4A). This correlated with morphological features in *Klf1*^−/−^ fetal liver erythroid cells, such as the presence of nucleated erythroid cells with F-actin protrusions or blebs, and small anucleate cell fragments rich in F-actin, resembling motile platelets (Bearer et al., 2002) (Fig. 4B).

### Loss of KLF1 leads to an increased presence of binucleate erythroblasts during terminal erythroid differentiation

**Fig. 5.**
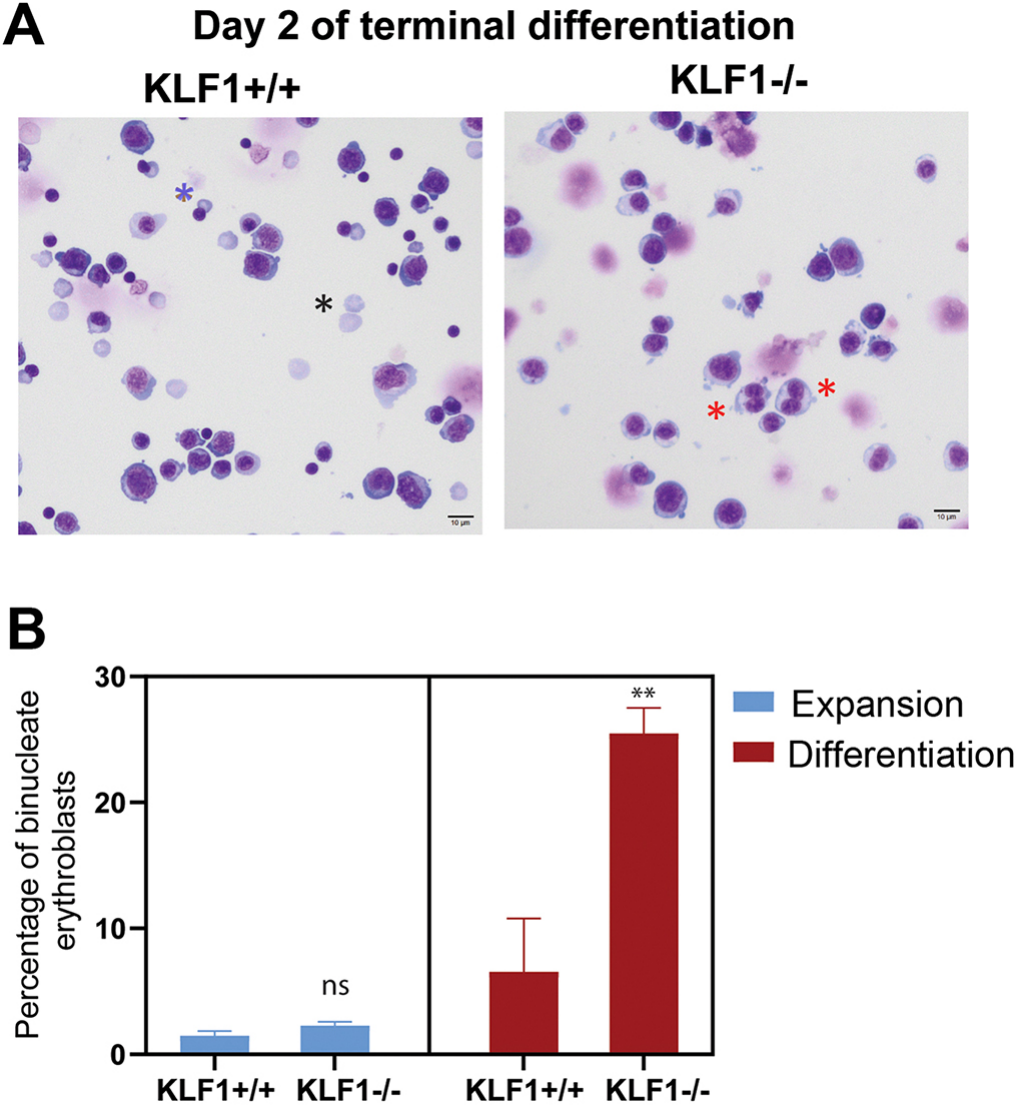
**There is a higher percentage of binucleate cells in *Klf1***^−/−^ **erythroid cells during terminal differentiation.** (A) Representative MGG-stained cytospins of *Klf1*^+/+^ and *Klf1*^−/−^ erythroid cells obtained from day 2 of terminal differentiation. Red asterisks indicate binucleate cells; black asterisk indicates enucleated cells; blue asterisk indicates enucleating orthochromatic erythroblasts. Scale bars: 10 µm. (B) Quantification of the MGG-stained cytopsins before and after terminal differentiation in *Klf1*^+/+^ and *Klf1*^−/−^ erythroid cultures show a higher incidence of binucleate erythroid cells in *Klf1*^−/−^ cultures during terminal differentiation compared to expansion (at least 100 cells from six fields from two biological replicates were quantified). Error bar represents s.e.m. **P≤0.02; ns, not significant (two-tailed, unpaired *t*-test).

To evaluate the consequence of the dysregulated cell division pathways in *Klf1*^−/−^ erythroblasts during terminal differentiation, we examined the morphology of *Klf1*^+/+^ and *Klf1*^−/−^ erythroblasts before and after terminal differentiation by Giemsa staining and using confocal fluorescence microscopy. We observed an increased presence of binucleated cells in *Klf1*^−/−^ compared to in *Klf1*^+/+^ cultures during terminal erythroid differentiation, as analyzed by cytospin and Giemsa staining (Fig. 5A). The presence of binucleate cells was not the result of *ex vivo* differentiation, as these cells were also observed in freshly isolated fetal liver cells, as indicated by the presence of two nuclei within a large cell with a membrane demarcated by the F-actin rich cortex (Fig. 4B). Notably, we did not observe a significant difference in binucleate cells between *Klf1*^+/+^ and *Klf1*^−/−^ cultures during the expansion phase prior to terminal differentiation (Fig. 5B). These morphological observations are consistent with the transcriptomics analysis showing that cell division pathways are dysregulated in the absence of KLF1 only during terminal differentiation.

### Loss of KLF1 leads to a failure in cytokinesis during murine terminal erythroid differentiation

**Fig. 6.**
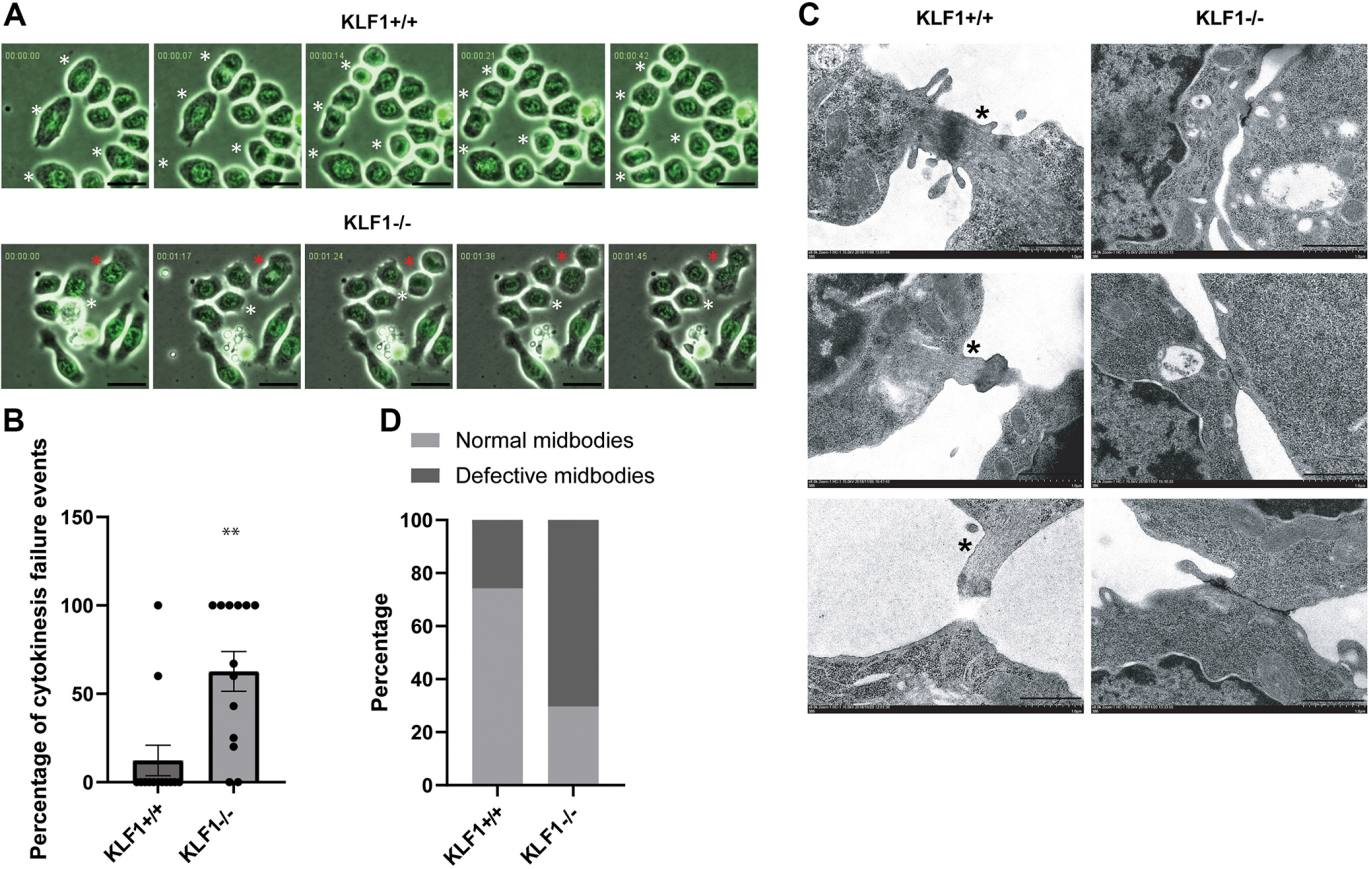
**Failure of cytokinesis during terminal erythroid differentiation.** (A) Representative frames of live-cell time-lapse images of histone-H2B–GFP-transduced *Klf1*^+/+^ and *Klf1*^−/−^ erythroblasts. Cells were imaged for 8 h every 7 min on day 2 of terminal differentiation. Red asterisks show regressed cytokinesis events and white asterisks show completed cytokinesis events. Merged images of bright field (whole cell) and GFP (nucleus) are shown (Movies 1–4; see also Fig. S3 for quantification and visualization of DNA bridges in these cells). Scale bars: 10 µm. (B) Quantification of cytokinesis failure events observed in live time-lapse imaging during terminal differentiation from 10 fields in two biological replicates in histone-H2B–GFP-transduced *Klf1*^+/+^ and *Klf1*^−/−^ cells are shown as a percentage of cytokinesis failure events among all cytokinesis events observed. Error bars represent s.e.m. **P≤0.005 (two-tailed, unpaired *t*-test). (C) Representative TEM images showing the presence of defective midbody architecture and impaired bundling of microtubules in cytokinesis bridges in *Klf1*^−/−^ erythroblasts, unlike *Klf1*^+/+^ erythroblasts (marked with asterisks), during day 2 of terminal differentiations. Scale bars: 1 µm. (D) Quantification of defective midbodies obtained from TEM imaging of 35 cytokinesis bridges imaged from *Klf1*^+/+^ erythroblasts and 37 cytokinesis bridges imaged from *Klf1*^−/−^ erythroblasts during day 2 of terminal differentiation.

To study the binucleate phenotype further in *Klf1*^−/−^ erythroblasts undergoing terminal differentiation, we employed live time-lapse imaging. For this, we transduced *Klf1*^+/+^ and *Klf1*^−/−^ erythroid cells with a histone-H2B–GFP expression vector to visualize the nucleus and used bright field to visualize the whole cell. *Klf1*^+/+^ and *Klf1*^−/−^ erythroblasts were imaged every 7 min for 8 h on day 2 of terminal differentiation. These studies showed that furrow formation and ingression during telophase proceeded normally in *Klf1*^−/−^ cells, but complete abscission of daughter cells failed (Fig. 6A–C). Remarkably, the cells that are in the process of separating ‘snap back’ together, yielding bi-nucleate cells (time-lapse videos available in Movies 1–4). Additional observations of the time-lapse images revealed the presence of DNA bridges only in the *Klf1*^−/−^ cells (Fig. S3). These amount to 23% of the failed cytokinesis events (∼8% of all cytokinesis events). Furthermore, electron microscopy analysis of cytokinesis bridges and midbodies (electron-dense region important for recruitment of proteins important for cytokinesis) revealed aberrant architecture and impaired microtubule bundling in *Klf1*^−/−^ erythroblasts (Fig. 6C,D). These results demonstrate a complete failure of terminal erythroid processes at multiple levels and explain the stalled, orthochromatic cell phenotype that cannot progress further.

Many genes involved in cytokinesis are regulated by KLF1 and fall into the category of genes whose induction during differentiation does not occur in the absence of KLF1 (Fig. S4). *Kif23* is a central spindle protein that is important for cytokinesis. Mutation in this gene causes CDA III in humans and its pathogenesis is characterized by a cytokinesis failure, and a high percentage of binucleate erythroblasts in the bone marrow (Gambale et al., 2016; Iolascon et al., 2013; Liljeholm et al., 2013). Citron kinase (*Cit*) is a contractile ring protein that is important for cytokinesis (Madaule et al., 1998; McKenzie et al., 2016). It has been shown that both Kif23 and Cit have strong KLF1-binding peaks in a previous ChIP-Seq study (Mukherjee and Bieker, 2022; Tallack et al., 2010, 2012). *Incenp* is a chromosomal passenger protein that regulates cytokinesis via activation of Aurora B kinase during abscission of daughter cells (Kaitna et al., 2000; Xu et al., 2009); it is very highly induced during differentiation but remains low in the absence of KLF1. Based on these studies, we can infer that KLF1 transcriptionally upregulates genes involved in cytokinesis during terminal erythroid cell divisions to accommodate the rapid pace of these divisions to undergo proper terminal differentiation.

Collectively, these studies suggest that the binucleate phenotype in *Klf1*^−/−^ erythroblasts arises due to a failure to complete abscission at cytokinesis bridges during cytokinesis. This follows from a deficient expression of component genes crucial for structural integrity of the cell to enable the progression of normal differentiation.

### Dysregulation of cell division pathways is similar in murine *Klf1*^−/−^ and human CDA IV erythroid cells during terminal differentiation

**Fig. 7.**
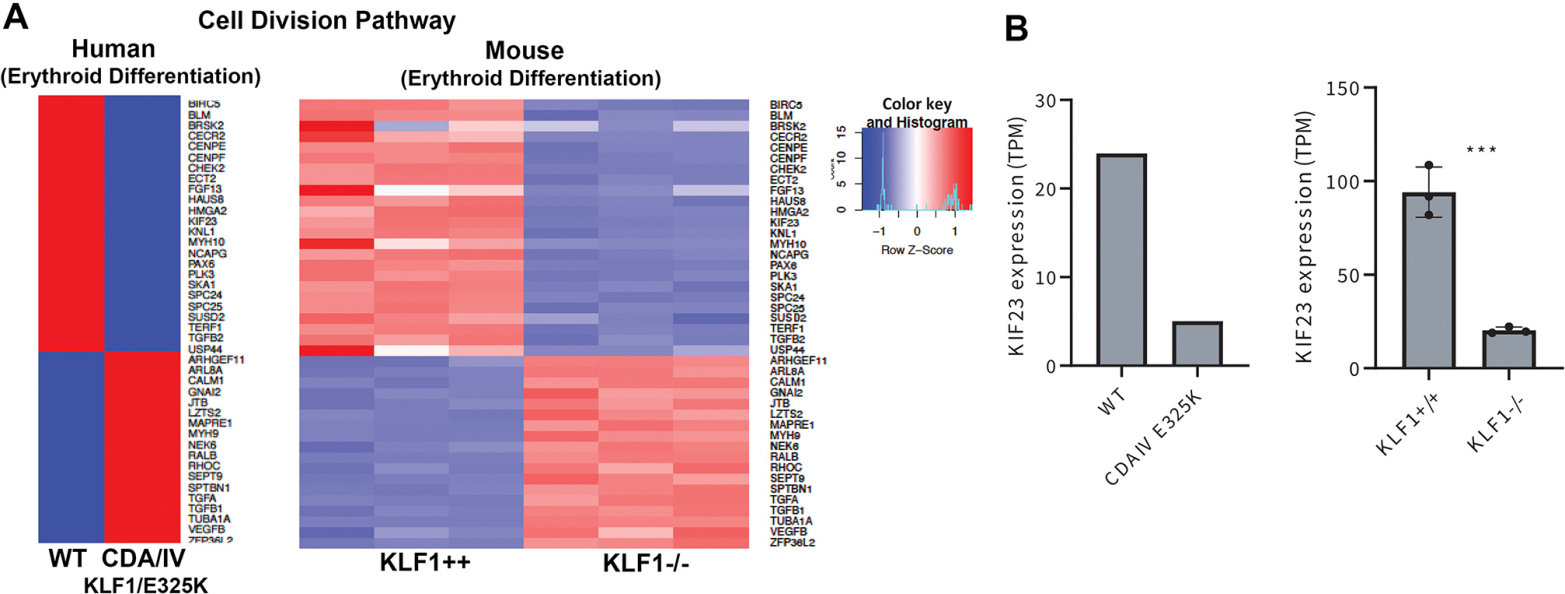
**Comparison of human and mouse RNA-seq datasets.** (A) Merging of RNA-Seq data from murine *Klf1*^+/+^ and *Klf1*^−/−^ erythroblasts (day 2 of differentiation) with that from erythroblasts from a human individual with CDA IV (KLF1/E325 heterozygous; day 5 of differentiation) (Varricchio et al., 2019) shows that cell division pathway has common gene signature of differentially expressed genes during terminal differentiation. Heat map for the cell division pathway is shown. (B) The normalized, relative gene expression [transcripts per million (TPM)] for KIF23 is shown for both human and murine differentiation datasets. Human data is from a control and a CDA IV individual; mouse data is from biological triplicates. Data is mean±s.e.m. ***P<0.001 (two-tailed, unpaired *t*-test).

A heterozygous KLF1 variant in the second zinc finger (E325K, glutamic acid to lysine) leads to CDA type IV (OMIM 613673) in humans (Bieker and Philipsen, 2024). The bone marrow of these individuals shows an increased presence of binucleate erythroid cells (Arnaud et al., 2010; Jaffray et al., 2013), similar to our observations in murine *Klf1*^−/−^ erythroid terminally differentiating cultures. To compare the transcriptional dysregulation in these two conditions, we merged our RNA-Seq data from differentiated murine *Klf1*^+/+^ and *Klf1*^−/−^ ESRE erythroid cells with the differentiated erythroid cell RNA-Seq data subset from an individual with CDA IV (Varricchio et al., 2019; GEO accession GSE128718). Cell division-, mitosis- and megakaryocyte differentiation-related GO pathways were dysregulated in both conditions (Fig. S5A). The pattern of the dysregulation in cell division GO pathway genes was conserved in both the murine *Klf1*^−/−^ and human CDA mutant erythroid cells (Fig. 7A). Similarly, extensive protein changes in cell cycle and chromatin separation networks were observed in proteomic analyses of the human BEL-A CDA IV cell model (Ferrer-Vicens et al., 2023). Of note, *KIF23* levels were decreased in both human and mouse samples (Fig. 7B), linking KLF1 function to the CDA type III phenotype. Furthermore, gene expression of *FLI1*, a megakaryocyte-specific gene that is normally repressed in erythroid cells, shows increased expression in the erythroid cells from a CDA IV individual (Fig. S5B) similar to the murine *Klf1*^−/−^ erythroid cells (Fig. 2C). Overall, these analyses provide a direct connection between the mouse and human data, and suggest that KLF1 is also playing a crucial role in establishing the proper gene expression context for human terminal differentiation, a process that can go awry in the presence of the dominant KLF1 CDA variant.

## DISCUSSION

Mammalian terminal erythroid development is a precisely regulated process involving rapid terminal cell divisions and serial morphological changes of erythroid progenitors to form mature erythrocytes (Lodish et al., 2010). In many somatic cells, terminal cell divisions associated with differentiation involve a transition in cell cycle dynamics, marked by an extension of the G1 phase, resulting in increased cell division time (Soufi and Dalton, 2016). However, erythroid cells exhibit a unique pattern, where commitment to terminal differentiation is accompanied by a shift from self-renewal divisions to rapid terminal cell divisions, termed ‘differentiation divisions’. These differentiation divisions are characterized by a shortened G1 phase and accelerated DNA replication (Dolznig et al., 1995; Pop et al., 2010; von Lindern, 2006; von Lindern et al., 2001). The regulatory mechanisms that govern the integrity of these rapid terminal divisions are not well understood. The current study suggests that cell division pathways undergo robust regulation by KLF1, an erythroid-enriched master transcriptional regulator, during terminal differentiation, possibly to accommodate the accelerated pace of erythroid terminal cell divisions.

Our study shows that KLF1 regulates cytokinesis and cell division pathways predominantly during terminal differentiation. Notably, genes important for these pathways are directly bound and upregulated by KLF1. Transcriptional dysregulation of these pathways correlates with the morphological phenotypes observed in *Klf1*^−/−^ erythroid cells, including an increased presence of binucleate erythroid cells during terminal erythroid differentiation. Live-cell time-lapse imaging revealed that these cells arise in *Klf1*^−/−^ cultures due to regression of the cytokinesis furrow and failure of abscission. Furthermore, electron microscopy analysis revealed that the cytokinesis bridges and midbodies in *Klf1*^−/−^ erythroblasts display aberrant architecture and defective microtubule bundling. Our investigation underscores stage-specific gene regulation of cell division and cytokinesis genes by KLF1 during erythropoiesis, likely to maintain the integrity of terminal cell divisions during terminal differentiation. The striking enrichment of cytokinesis and cell division pathways specifically during terminal differentiation and only in genes bound and upregulated by KLF1 suggests that KLF1 acts as an activator to induce the expression of its target genes involved in cell division during terminal erythroid differentiation. In addition, and in agreement with previous studies, megakaryocyte differentiation related pathways and genes (e.g. *FLI1*) are upregulated in *Klf1*^−/−^ erythroid cells (Bouilloux et al., 2008; Frontelo et al., 2007); this dysregulation correlates with megakaryocyte-like morphological phenotypes observed in *Klf1*^−/−^ erythroid cells, for example F-actin-rich protrusions and anucleate cell fragments (Bearer et al., 2002).

Understanding the regulation of cell division pathways during erythroid terminal differentiation has direct relevance to advancing our comprehension of the pathogenesis of CDAs. CDAs are characterized by severe molecular defects in DNA replication and cytokinesis, which, in turn, lead to impaired erythroid terminal divisions and the distinctive pathological phenotype of CDAs (Gambale et al., 2016; Iolascon et al., 2013). This phenotype includes a high prevalence of binucleated erythroblasts, sometimes accompanied by DNA bridges (Gambale et al., 2016; Iolascon et al., 2013). The perplexing aspect of these disorders is how mutations in genes that typically play ubiquitous roles in regulating DNA replication and cytokinesis affect the erythroid lineage selectively while largely sparing other lineages. CDA IV arises due to a heterozygous mutation E325K in the second zinc finger of KLF1 (Kulczynska-Figurny et al., 2020), and results in severe anemia characterized by an increased prevalence of binucleate erythroid cells in the bone marrow (Arnaud et al., 2010; Jaffray et al., 2013; Singleton et al., 2009, 2011). Previous studies have shown that these phenotypes are a result of both ectopic binding by the mutant protein and impairment of wild-type KLF1 binding to its targets (Ferrer-Vicens et al., 2023; Ilsley et al., 2019; Kulczynska et al., 2020; Varricchio et al., 2019). In the current study, a comparison of transcriptomic data from murine *Klf1*^−/−^ erythroblasts and human CDA IV erythroblasts (harboring the heterozygous KLF1 E325K mutation) revealed that a similar subset of cell division genes are dysregulated during terminal differentiation. These studies suggest that the binding of KLF1 to gene targets in cell division pathways is impaired due to the presence of the mutant CDA allele in the CDA IV disease context.

The characterization of the cytokinesis defects in *Klf1*^−/−^ erythroid cells show a failure in abscission due to regression of the cytokinesis furrow. A close examination of the midbody region and cytokinesis bridge using electron microscopy showed that the overall architecture and microtubule bundling are impaired. Interestingly KIF23 mutations also give rise to these similar phenotypes and pathogenic *KIF23* variants cause CDA type III in humans (Gambale et al., 2016; Iolascon et al., 2013; Liljeholm et al., 2013). Our studies identify KIF23 as a direct target of KLF1 that is dysregulated in both murine *Klf1*^−/−^ and in human CDA IV erythroblasts during terminal differentiation (Varricchio et al., 2019). The extent to which KIF23 deficiency contributes to the observed cytokinesis defects, and whether these defects can be rescued by expression of this gene in murine *Klf1*^−/−^ and human CDA IV erythroblasts, warrants further investigations.

In summary, by studying the dynamics of pathways and genes regulated by KLF1 before and after mammalian terminal erythroid differentiation, our studies using primary cells derived from WT or KLF1-KO mice have illuminated the molecular and regulatory mechanisms that explain why KLF1 is necessary for the final stages of terminal erythropoiesis. These data show that defective cell biology and structural deficits follow from erythroid cells that do not express KLF1. Phenotypically, these are exemplified by cytokinesis failure, the presence of binucleate cells and intranuclear DNA bridges, as well as aberrant cell protrusions and fragmentation. Most of the relevant genes are direct binding targets of KLF1. As a result, these downstream effects can be added to the known cell cycle deficiencies also apparent when KLF1 is not expressed. These data are directly relevant to human CDA, particularly type IV, which also exhibits an extensive level of binucleate forms and DNA bridges, and that is also defective in expression of relevant cytokinesis genes.

## MATERIALS AND METHODS

### Cell culture

Extensively self-renewing erythroblasts from *Klf1*^+/+^ and *Klf1*^−/−^ E12.5 mouse fetal livers were expanded and terminally differentiated using protocols as described previously (England et al., 2011; Gnanapragasam et al., 2016). These cells from each embryo provided a biological replicate for analysis. Embryos were from 4–6-week-old heterozygous *Klf1*^+/−^ females obtained after mating with heterozygous *Klf1*^+/−^ males (Perkins et al., 1995). All are in C57BL6 background. Animal experiments were approved by the IACUC committee at Mount Sinai, protocol 2019-0042.

### MGG staining

May–Grünwald Giemsa (MGG) staining was performed as described previously. The slides were imaged using Nikon Eclipse Ts100 microscope with a 40× objective. Images were acquired using ProgRes Mac CapturePro 2.7 software. All scale bars shown are 10 µm in length.

### RNA sequencing and analysis

RNA isolation was performed as previously described using Tri reagent (Sigma) (Elagooz et al., 2022). A poly(A) library was prepared and HiSeq 100 nt single-read sequencing was performed to obtain greater than 40 million reads per sample at the Institute for Genomics and Multiscale Biology at the Icahn School of Medicine at Mount Sinai, New York, USA. RNA isolation, library formation and analyses for all samples were performed at the same time in the same way. The number of total reads for each of the 12 samples were within ±18.5% of the average for all, and the number of total genes and transcripts detected were within ±3.8% across all 12 samples.

### RNA-seq data analysis

The mouse RNA-seq reads were mapped to the mouse genes (Ensembl v75) using Bowtie (v0.12.8) (Langmead et al., 2009) allowing up to two mismatches and a maximum of 200 multiple hits. The gene expected read counts and transcripts per million (TPMs) were estimated by RSEM (v1.2.3; Li and Dewey, 2011). TPM counts used for reporting RNA-seq results represent the relative abundance of transcripts after normalization for transcript length, and do not directly reflect absolute copy numbers per cell. As a result, we have not made any conclusions regarding RNA expression per cell or absolute levels. TPMs were median-by-ratio normalized, and replicates were merged via calculating average normalized TPMs. The derived normalized TPM data used for the figures are listed in Table S1. To determine differentially expressed genes (DEGs), the EBSeq package (Leng et al., 2013) was used to assess the probability of gene expression (mRNAs) being differentially expressed between any two given conditions. We required that DEGs should have a false discovery rate (FDR)<5% via EBSeq and >2-fold-change of ‘normalized read counts+1’. EBSeq is a robust approach for analysis of sample sizes as small as three for each group (Li et al., 2022), such as the case here. In addition, EBSeq has a higher statistical power than other analysis programs when comparing and identifying DEGs (Leng et al., 2013; Li et al., 2022). Using these criteria, analysis of a number of housekeeping genes (*Gapdh*, *Actb*, *Rpl32*, *Rpl27*, *B2m* and *Sdha*) show insignificant changes in expression level between expansion and differentiation stages (Fig. S1B) as also seen with nearly half of the genes queried (Fig. 2A). Gene Ontology (GO) enrichment analysis: Gene ontology (GO) enrichment analysis was performed using the R package (‘allez’) (Newton et al., 2007). The enrichment *P*-values were further adjusted by Benjamini–Hochberg (BH) multiple test correction. This data has been deposited in GEO (https://www.ncbi.nlm.nih.gov/geo/).

### Integration of ChIP-seq with RNA-seq data

To identify differentially expressed genes that are potentially regulated by transcription factor binding, we integrated RNA-seq transcriptomic profiles with previous ChIP-seq peak data (Ilsley et al., 2019; GEO accession GSE92620). For each transcript identified by RNA-seq, we defined a cis-regulatory window spanning ±2 kilobases (kb) from the annotated transcription start site (TSS). ChIP-seq peaks overlapping this 4 kb window were interpreted as evidence of potential transcriptional regulation by the TF. Only differentially expressed genes with ChIP-seq peak overlap within this window were defined as ChIP-seq targets and selected for downstream analysis.

### Live time-lapse imaging

Erythroid cells were transduced with lentivirus generated using Histone-H2B–GFP lentiviral vector (a generous gift from Dr Emily Bernstein, Icahn School of Medicine at Mount Sinai, New York, USA) (Gaspar-Maia et al., 2013) and FACS-sorted for the GFP-positive cells. We then plated these cells on recombinant fibronectin (Takara) coated plates for live time-lapse imaging. Imaging was done using an Olympus IX-70 with LiveCell microscope, for a period of 8 h, every 7 min, after 24 h of differentiation. We imaged ten fields for each biological replicate of *Klf1*^+/+^ and *Klf1*^−/−^ erythroid cells. The scale bar represents 10 µm in length.

### Transmission electron microscopy imaging

Cells were grown on Lab-Tek Permanox chamber slides (EMS) with differentiation medium (Gnanapragasam et al., 2016), and on day 2 of erythroid terminal differentiation, cells were washed with sodium cacodylate buffer and fixed with 2% paraformaldehyde and 2–2.5% glutaraldehyde in 0.1 M sodium cacodylate buffer (pH 7.2–7.4) for 24 h before processing for epoxy resin embedding. Samples were fixed with 1.5% potassium ferricyanide, 2% osmium tetroxide, 0.1 M sodium cacodylate (pH 7.2–7.4) buffer [Electron microscopy Sciences (EMS), Hatfield, PA, USA] followed with an *en bloc* staining of 2% uranyl acetate. Samples were dehydrated via graded ethanol series (25%, 50%, 70%, 95%, 100%) and then infiltrated with propylene oxide and ‘Embed 812’ Epon resin (EMS). Tissue was placed in BEEM embedding capsule molds (EMS), filled with resin and heat polymerized at 60°C for 72 h in a vacuum oven. Semi-thin sections (0.5 μm and 1 μm) were obtained using a Leica UC7 ultramicrotome (Leica Biosystems Inc., Buffalo Grove, IL, USA), counter-stained with 1% Toluidine Blue, cover slipped and viewed under a light microscope to identify and secure the region of interest. Ultra-thin sections (85 nm) were cut with a diamond knife (Diatome, Hatfield, PA, USA) and collected on copper 300 mesh grids (EMS) using a Coat-Quick adhesive pen. Sections were counter-stained with 1% uranyl acetate followed with lead citrate. Imaging was undertaken using a HT7500 transmission electron microscope (Hitachi High-Technologies, Tokyo, Japan) using an AMT NanoSprint12 12-megapixel CMOS TEM Camera (Advanced Microscopy Techniques, Danvers, MA, USA).

### Immunostaining and confocal fluorescence microscopy

Fetal livers from *Klf1*^+/+^ or *Klf1*^−/−^ E12.5 embryos were dissociated into PBS by gentle trituration using a cut-off P200 tip, followed by overnight fixation in 4% paraformaldehyde (PFA) in PBS at 4°C. Cells were washed in PBS several times, centrifuged in an Eppendorf microfuge at 1500 ***g*** for 4 min, re-suspended in permeabilization buffer (0.3% Triton X-100 in PBS), and incubated for 20 min at room temperature. Cells were stored in blocking buffer (3% BSA and 1% goat serum in PBS) at least overnight at 4°C. Blocked cells were stained for 2 h at room temperature with primary Alexa Fluor 488-conjugated rat anti-Ter119 antibody (1:100, BioLegend, #116215), Rhodamine–phalloidin (1:100, Invitrogen, #R415) to stain F-actin, and Hoechst 33258 (1:1000, Invitrogen, #H1398) to stain nuclei; all diluted in blocking buffer. Cells were then deposited onto slides using a Thermo Scientific Cytospin 4 cytocentrifuge at 112 ***g*** for 3 min, coverslipped with Fluoro-Gel (Electron Microscopy Sciences, Hatfield, PA), and imaged within 7 days. Images were acquired on a Zeiss LSM 780 laser scanning confocal microscope with a 100×/1.4 N.A. objective using zoom 2. For *Z*-stacks, the step size was 0.3 µm, and enough *Z*-steps were collected to image the whole cell (usually between 17 and 25 steps). Images were processed using Volocity 6.1.1 and Adobe Photoshop, and image figures were constructed in Adobe Illustrator.

### Western blotting

As described previously (Gnanapragasam et al., 2016), cells were lysed using RIPA buffer and lysates were resolved using a 4–20% gradient SDS-PAGE gel (Bio-Rad), and blotted onto a PVDF membrane. Protein levels were probed using anti-EKLF antibody [1:1000; 7B2a antibody, manufactured in-house, characterized in reference Mukherjee and Bieker (2022)] and anti-GAPDH antibody (1:1000; Sigma, #G8795, clone GAPDH-71.1, RRID: AB_1078991), followed by goat anti-mouse IgG(H+L)-HRP secondary antibody (1:5000; Thermo Fisher Scientific, #31430). Uncropped images of western blots from this paper are shown in Fig. S6.

## Supplementary Material

10.1242/joces.264036_sup1 Supplementary information

Table S1. Normalized TPM. Listed are all TPM values for the 12 samples analyzed, including triplicates of WT and *Klf1-/-* erythroid cells, each under expansion (amplifying) and differentiating conditions. Processing of the raw data was as described in Materials and Methods. These data form the basis for the graphs in Figs. 1-4, S1, S2, S4, S5.

Table S2. Principle component analysis. x- and y-coordinate values for the data in Fig. 1B are shown.

## References

[JCS264036C1] Arnaud, L., Saison, C., Helias, V., Lucien, N., Steschenko, D., Giarratana, M.-C., Prehu, C., Foliguet, B., Montout, L., de Brevern, A. G. et al. (2010). A dominant mutation in the gene encoding the erythroid transcription factor KLF1 causes a congenital dyserythropoietic anemia. *Am. J. Hum. Genet.* 87, 721-727. 10.1016/j.ajhg.2010.10.010 21055716 PMC2978953

[JCS264036C2] Bearer, E. L., Prakash, J. M. and Li, Z. (2002). Actin dynamics in platelets. *Int. Rev. Cytol.* 217, 137-182. 10.1016/S0074-7696(02)17014-8 12019562 PMC3376087

[JCS264036C3] Bieker, J. J. and Philipsen, S. (2024). Erythroid Krüppel-Like Factor (KLF1): a surprisingly versatile regulator of erythroid differentiation. *Adv. Exp. Med. Biol.* 1459, 217-242. 10.1007/978-3-031-62731-6_10 39017846 PMC12121306

[JCS264036C4] Bouilloux, F., Juban, G., Cohet, N., Buet, D., Guyot, B., Vainchenker, W., Louache, F. and Morlé, F. (2008). EKLF restricts megakaryocytic differentiation at the benefit of erythrocytic differentiation. *Blood* 112, 576-584. 10.1182/blood-2007-07-098996 18523154

[JCS264036C5] Chen, K., Liu, J., Heck, S., Chasis, J. A., An, X. and Mohandas, N. (2009). Resolving the distinct stages in erythroid differentiation based on dynamic changes in membrane protein expression during erythropoiesis. *Proc. Natl. Acad. Sci. USA* 106, 17413-17418. 10.1073/pnas.0909296106 19805084 PMC2762680

[JCS264036C6] Dolznig, H., Bartunek, P., Nasmyth, K., Müllner, E. W. and Beug, H. (1995). Terminal differentiation of normal chicken erythroid progenitors: shortening of G1 correlates with loss of D-cyclin/cdk4 expression and altered cell size control. *Cell Growth Differ.* 6, 1341-1352. 8562472

[JCS264036C7] Elagooz, R., Dhara, A. R., Gott, R. M., Adams, S. E., White, R. A., Ghosh, A., Ganguly, S., Man, Y., Owusu-Ansah, A., Mian, O. Y. et al. (2022). PUM1 mediates the posttranscriptional regulation of human fetal hemoglobin. *Blood Adv*. 6, 6016-6022. 10.1182/bloodadvances.2021006730 35667093 PMC9699939

[JCS264036C8] England, S. J., McGrath, K. E., Frame, J. M. and Palis, J. (2011). Immature erythroblasts with extensive ex vivo self-renewal capacity emerge from the early mammalian fetus. *Blood* 117, 2708-2717. 10.1182/blood-2010-07-299743 21127173 PMC3062358

[JCS264036C9] Ferrer-Vicens, I., Ferguson, D. C. J., Wilson, M. C., Heesom, K. J., Bieker, J. J. and Frayne, J. (2023). A novel human cellular model of CDA IV enables comprehensive analysis revealing the molecular basis of the disease phenotype. *Blood* 141, 3039-3054. 10.1182/blood.2022018735 37084386 PMC10315626

[JCS264036C10] Frontelo, P., Manwani, D., Galdass, M., Karsunky, H., Lohmann, F., Gallagher, P. G. and Bieker, J. J. (2007). Novel role for EKLF in megakaryocyte lineage commitment. *Blood* 110, 3871-3880. 10.1182/blood-2007-03-082065 17715392 PMC2190608

[JCS264036C11] Gambale, A., Iolascon, A., Andolfo, I. and Russo, R. (2016). Diagnosis and management of congenital dyserythropoietic anemias. *Expert Rev. Hematol.* 9, 283-296. 10.1586/17474086.2016.1131608 26653117

[JCS264036C12] Gaspar-Maia, A., Qadeer, Z. A., Hasson, D., Ratnakumar, K., Adrian Leu, N., Leroy, G., Liu, S., Costanzi, C., Valle-Garcia, D., Schaniel, C. et al. (2013). MacroH2A histone variants act as a barrier upon reprogramming towards pluripotency. *Nat. Commun.* 4, 1565. 10.1038/ncomms2582 23463008 PMC4055026

[JCS264036C13] Gnanapragasam, M. N. and Bieker, J. J. (2017). Orchestration of late events in erythropoiesis by KLF1/EKLF. *Curr. Opin. Hematol.* 24, 183-190. 10.1097/MOH.0000000000000327 28157724 PMC5523457

[JCS264036C14] Gnanapragasam, M. N., McGrath, K. E., Catherman, S., Xue, L., Palis, J. and Bieker, J. J. (2016). EKLF / KLF1-regulated cell cycle exit is essential for erythroblast enucleation. *Blood* 128, 1631-1641. 10.1182/blood-2016-03-706671 27480112 PMC5034741

[JCS264036C15] Ilsley, M. D., Huang, S., Magor, G. W., Landsberg, M. J., Gillinder, K. R. and Perkins, A. C. (2019). Corrupted DNA-binding specificity and ectopic transcription underpin dominant neomorphic mutations in KLF / SP transcription factors. *BMC Genomics* 20, 417. 10.1186/s12864-019-5805-z 31126231 PMC6534859

[JCS264036C16] Iolascon, A., Heimpel, H., Wahlin, A. and Tamary, H. (2013). Congenital dyserythropoietic anemias: molecular insights and diagnostic approach. *Blood* 122, 2162-2166. 10.1182/blood-2013-05-468223 23940284 PMC3785118

[JCS264036C17] Isern, J., Fraser, S. T., He, Z., Zhang, H. and Baron, M. H. (2010). Dose-dependent regulation of primitive erythroid maturation and identity by the transcription factor Eklf. *Blood* 116, 3972-3980. 10.1182/blood-2010-04-281196 20720183 PMC2981545

[JCS264036C18] Jaffray, J. A., Mitchell, W. B., Gnanapragasam, M. N., Seshan, S. V., Guo, X., Westhoff, C. M., Bieker, J. J. and Manwani, D. (2013). Erythroid transcription factor EKLF/KLF1 mutation causing congenital dyserythropoietic anemia type IV in a patient of Taiwanese origin: review of all reported cases and development of a clinical diagnostic paradigm. *Blood Cells Mol. Dis.* 51, 71-75. 10.1016/j.bcmd.2013.02.006 23522491 PMC4560093

[JCS264036C19] Kaitna, S., Mendoza, M., Jantsch-Plunger, V. and Glotzer, M. (2000). Incenp and an Aurora-like kinase form a complex essential for chromosome segregation and efficient completion of cytokinesis. *Curr. Biol.* 10, 1172-1181. 10.1016/S0960-9822(00)00721-1 11050385

[JCS264036C20] Kulczynska-Figurny, K., Bieker, J. J. and Siatecka, M. (2020). Mutation research / reviews in mutation research severe anemia caused by dominant mutations in Krüppel-like factor 1 (KLF1). *Mutat. Res. Mutat. Res.* 786, 108336. 10.1016/j.mrrev.2020.108336 PMC10199782 33339573

[JCS264036C21] Kulczynska, K., Bieker, J. J. and Siatecka, M. (2020). A Krüppel-like factor 1 (KLF1) mutation associated with severe congenital dyserythropoietic anemia alters its DNA-binding specificit. *Mol. Cell. Biol.* 40, e00444-19. 10.1128/MCB.00444-19 31818881 PMC7020642

[JCS264036C22] Langmead, B., Trapnell, C., Pop, M. and Salzberg, S. L. (2009). Ultrafast and memory-efficient alignment of short DNA sequences to the human genome. *Genome Biol.* 10, R25. 10.1186/gb-2009-10-3-r25 19261174 PMC2690996

[JCS264036C23] Leng, N., Dawson, J. A., Thomson, J. A., Ruotti, V., Rissman, A. I., Smits, B. M. G., Haag, J. D., Gould, M. N., Stewart, R. M. and Kendziorski, C. (2013). EBSeq: an empirical Bayes hierarchical model for inference in RNA-seq experiments. *Bioinformatics* 29, 1035-1043. 10.1093/bioinformatics/btt087 23428641 PMC3624807

[JCS264036C24] Li, B. and Dewey, C. N. (2011). RSEM: accurate transcript quantification from RNA-Seq data with or without a reference genome. *BMC Bioinformatics* 12, 23. 10.1186/1471-2105-12-323 21816040 PMC3163565

[JCS264036C25] Li, D., Zand, M. S., Dye, T. D., Goniewicz, M. L., Rahman, I. and Xie, Z. (2022). An evaluation of RNA-seq differential analysis methods. *PLoS ONE* 17, e0264246. 10.1371/journal.pone.0264246 36112652 PMC9480998

[JCS264036C26] Liljeholm, M., Irvine, A. F., Vikberg, A. L., Norberg, A., Month, S., Sandström, H., Wahlin, A., Mishima, M. and Golovleva, I. (2013). Congenital dyserythropoietic anemia type III (CDA III) is caused by a mutation in kinesin family member, KIF23. *Blood* 121, 4791-4799. 10.1182/blood-2012-10-461392 23570799

[JCS264036C27] Lodish, H., Flygare, J. and Chou, S. (2010). From stem cell to erythroblast: regulation of red cell production at multiple levels by multiple hormones. *IUBMB Life* 62, 492-496. 10.1002/iub.322 20306512 PMC2893266

[JCS264036C28] Madaule, P., Eda, M., Watanabe, N., Fujisawa, K., Matsuoka, T., Bito, H., Ishizaki, T. and Narumiya, S. (1998). Role of citron kinase as a target of the small GTPase Rho in cytokinesis. *Nature* 394, 491-494. 10.1038/28873 9697773

[JCS264036C29] McKenzie, C., Bassi, Z. I., Debski, J., Gottardo, M., Callaini, G., Dadlez, M. and D'Avino, P. P. (2016). Cross-regulation between Aurora B and Citron kinase controls midbody architecture in cytokinesis. *Open Biol.* 6, 160019. 10.1098/rsob.160019 27009191 PMC4821246

[JCS264036C30] Miller, I. J. and Bieker, J. J. (1993). A novel, erythroid cell-specific murine transcription factor that binds to the CACCC element and is related to the Krüppel family of nuclear proteins. *Mol. Cell. Biol.* 13, 2776-2786. 10.1128/mcb.13.5.2776-2786.1993 7682653 PMC359658

[JCS264036C31] Mukherjee, K. and Bieker, J. J. (2022). EKLF/Klf1 regulates erythroid transcription by its pioneering activity and selective control of RNA Pol II pause-release. *Cell Rep.* 41, 111830. 10.1016/j.celrep.2022.111830 36543143 PMC9879271

[JCS264036C32] Newton, M. A., Quintana, F. A., den Boon, J. A., Sengupta, S. and Ahlquist, P. (2007). Random-set methods identify distinct aspects of the enrichment signal in gene-set analysis. *Ann. Appl. Stat.* 1, 85-106. 10.1214/07-AOAS104

[JCS264036C33] Newton, L. M., Fowler, V. M. and Humbert, P. O. (2024). Erythroblast enucleation at a glance. *J. Cell Sci.* 137, jcs261673. 10.1242/jcs.261673 39397781 PMC11529606

[JCS264036C34] Nuez, B., Michalovich, D., Bygrave, A., Ploemacher, R. and Grosveld, F. (1995). Defective haematopoiesis in fetal liver resulting from inactivation of the EKLF gene. *Nature* 375, 316-318. 10.1038/375316a0 7753194

[JCS264036C35] Perkins, A. C., Sharpe, A. H. and Orkin, S. H. (1995). Lethal β-thalassaemia in mice lacking the erythroid CACCC-transcription factor EKLF. *Nature* 375, 318-322. 10.1038/375318a0 7753195

[JCS264036C36] Perkins, A., Xu, X., Higgs, D. R., Patrinos, G. P., Arnaud, L., Bieker, J. J. and Philipsen, S. (2016). Kruppeling erythropoiesis : an unexpected broad spectrum of human red blood cell disorders due to KLF1 variants. *Blood* 127, 1856-1862. 10.1182/blood-2016-01-694331 26903544 PMC4832505

[JCS264036C37] Pilon, A. M., Arcasoy, M. O., Dressman, H. K., Vayda, S. E., Maksimova, Y. D., Sangerman, J. I., Gallagher, P. G. and Bodine, D. M. (2008). Failure of terminal erythroid differentiation in EKLF-deficient mice is associated with cell cycle perturbation and reduced expression of E2F2. *Mol. Cell. Biol.* 28, 7394-7401. 10.1128/MCB.01087-08 18852285 PMC2593440

[JCS264036C38] Pop, R., Shearstone, J. R., Shen, Q., Liu, Y., Hallstrom, K., Koulnis, M., Gribnau, J. and Socolovsky, M. (2010). A key commitment step in erythropoiesis is synchronized with the cell cycle clock through mutual inhibition between PU.1 and S-phase progression. *PLoS Biol.* 8, e1000484. 10.1371/journal.pbio.1000484 20877475 PMC2943437

[JCS264036C39] Shearstone, J. R., Pop, R., Bock, C., Boyle, P., Meissner, A. and Socolovsky, M. (2010). Mouse erythropoiesis in Vivo. *Landscape* 8. 10.1126/science.1207306 PMC3230325 22076376

[JCS264036C40] Siatecka, M. and Bieker, J. J. (2011). The multifunctional role of EKLF/KLF1 during erythropoiesis. *Blood* 118, 2044-2054. 10.1182/blood-2011-03-331371 21613252 PMC3292426

[JCS264036C41] Singleton, B. K., Fairweather, V. S., Lau, W., Parsons, S. F., Burton, N. M., Frayne, J., Brady, R. L. and Anstee, D. J. (2009). A novel EKLF mutation in a patient with dyserythropoietic anemia: the first association of EKLF with disease in man. *Blood* 114, 162. 10.1182/blood.V114.22.162.162

[JCS264036C42] Singleton, B. K., Lau, W., Fairweather, V. S. S., Burton, N. M., Wilson, M. C., Parsons, S. F., Richardson, B. M., Trakarnsanga, K., Brady, R. L., Anstee, D. J. et al. (2011). Mutations in the second zinc finger of human EKLF reduce promoter affinity but give rise to benign and disease phenotypes. *Blood* 118, 3137-3145. 10.1182/blood-2011-04-349985 21778342

[JCS264036C43] Soufi, A. and Dalton, S. (2016). Cycling through developmental decisions: how cell cycle dynamics control pluripotency, differentiation and reprogramming. *Development* 143, 4301-4311. 10.1242/dev.142075 27899507 PMC5201050

[JCS264036C44] Starck, J., Cohet, N., Gonnet, C., Sarrazin, S., Doubeikovskaia, Z., Doubeikovski, A., Verger, A., Duterque-Coquillaud, M. and Morle, F. (2003). Functional Cross-Antagonism between Transcription Factors FLI-1 and EKLF. *Mol. Cell. Biol.* 23, 1390-1402. 10.1128/MCB.23.4.1390-1402.2003 12556498 PMC141137

[JCS264036C45] Tallack, M. R. and Perkins, A. C. (2010). Megakaryocyte-erythroid lineage promiscuity in EKLF null mouse blood. *Haematologica* 95, 144-147. 10.3324/haematol.2009.010017 19850899 PMC2805734

[JCS264036C46] Tallack, M. R., Whitington, T., Yuen, W. S., Wainwright, E. N., Keys, J. R., Gardiner, B. B., Nourbakhsh, E., Cloonan, N., Grimmond, S. M., Bailey, T. L. et al. (2010). A global role for KLF1 in erythropoiesis revealed by ChIP-seq in primary erythroid cells. *Genome Res.* 20, 1052-1063. 10.1101/gr.106575.110 20508144 PMC2909569

[JCS264036C47] Tallack, M. R., Magor, G. W., Dartigues, B., Sun, L., Huang, S., Fittock, J. M., Fry, S. V., Glazov, E. A., Bailey, T. L. and Perkins, A. C. (2012). Novel roles for KLF1 in erythropoiesis revealed by mRNA-seq. *Genome Res.* 22, 2385-2398. 10.1101/gr.135707.111 22835905 PMC3514668

[JCS264036C48] Varricchio, L., Planutis, A., Manwani, D., Jaffray, J., Beau Mitchell, W., Migliaccio, A. R. and Bieker, J. J. (2019). Genetic disarray follows mutant KLF1-E325K expression in a congenital dyserythropoietic anemia patient. *Haematologica* 104, 2372-2380. 10.3324/haematol.2018.209858 30872368 PMC6959163

[JCS264036C49] von Lindern, M. (2006). Cell-cycle control in erythropoiesis. *Blood* 108, 781-782. 10.1182/blood-2006-05-022368

[JCS264036C50] von Lindern, M., Deiner, E. M. and Dolznig, H., Parren-van Amelsvoort, M., Hayman, M. J., Mullner, E. W. and Beug, H. (2001). Leukemic transformation of normal murine erythroid progenitors: v- and c-ErbB act through signaling pathways activated by the EpoR and c-Kit in stress erythropoiesis. *Oncogene* 20, 3651-3664. 10.1038/sj.onc.1204494 11439328

[JCS264036C51] Waye, J. S. and Eng, B. (2015). Krüppel-like factor 1: hematologic phenotypes associated with KLF1 gene mutations. *Int. J. Lab. Hematol.* 37, 78-84. 10.1111/ijlh.12356 25976964

[JCS264036C52] Xu, Z., Ogawa, H., Vagnarelli, P., Bergmann, J. H., Hudson, D. F., Ruchaud, S., Fukagawa, T., Earnshaw, W. C. and Samejima, K. (2009). INCENP-aurora B interactions modulate kinase activity and chromosome passenger complex localization. *J. Cell Biol.* 187, 637-653. 10.1083/jcb.200906053 19951914 PMC2806593

