# Supplementary material for: KLF1 coordinates specialized transcriptional networks required to maintain the integrity of terminal erythropoiesis
Source: J Cell Sci. 2025 Nov 13;138(21):jcs264036. doi: 10.1242/jcs.264036 (PMC12669967; doi:10.1242/jcs.264036)
Supplement: Supplementary information [file joces-138-264036-s1.pdf]

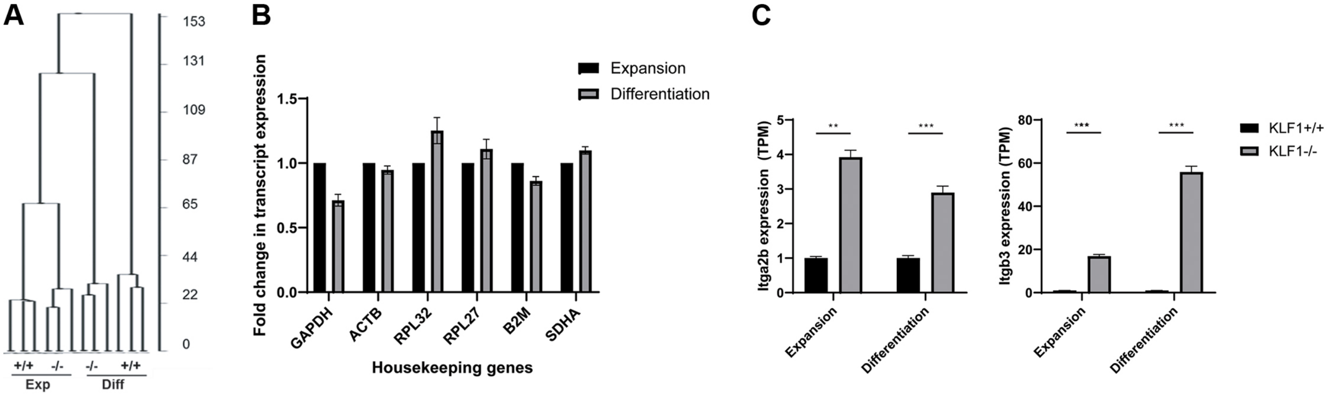

Fig. S1. RNA expression comparisons. (A) Hierarchical clustering analysis for RNA seq samples. Results are shown for triplicate samples from expanding and differentiating (d2) ESREs, from WT (+/+) or KO (-/-) sources. (B) Gene expression of housekeeping genes before and after differentiation in *Klf1*+/+ cells. (C) Gene expression of *Itga2b* and *Itgb3*, megakaryocyte-specific genes that are normally repressed in erythroid cells, show increased expression in both expanding and differentiating *Klf1*-/- cells compared to *Klf1*+/+ cells. Data is from biological triplicates analyzed for each condition. *** p<0.001; ** p ≤ 0.02

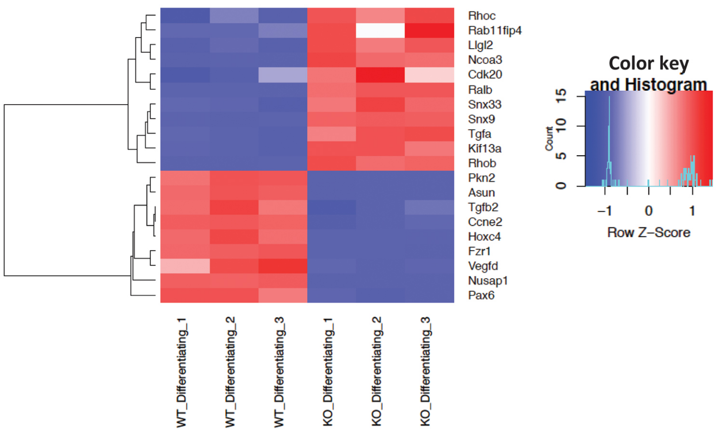

Fig. S2. The top 20 differentially regulated genes related to the cell division pathway. Triplicate samples from WT (+/+) and KO (-/-) cells were harvested after differentiation and compared for RNA expression of selected cell division pathway genes. Histogram (right) shows the color key for Z-score.

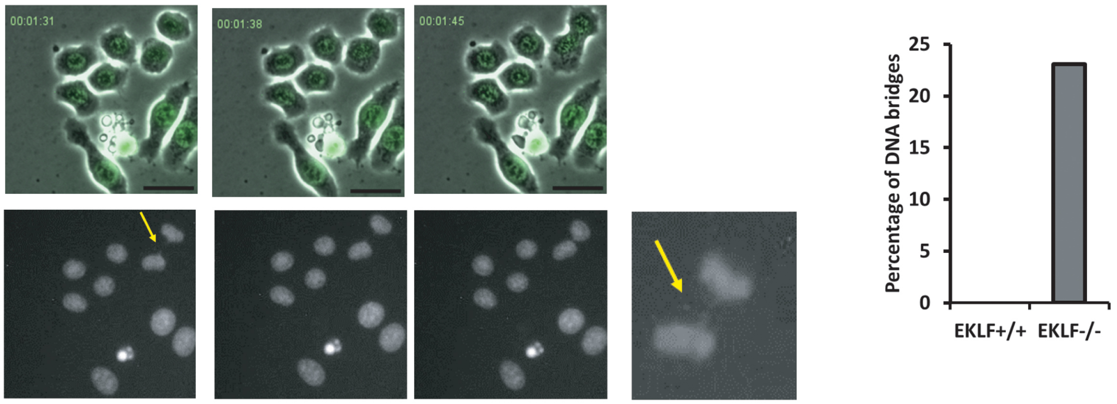

Fig. S3. Visualization and quantification of DNA bridges. *Left* shows brightfield (top) and immunofluorescent (bottom) images of differentiating *Klf1* -/- cells; arrow points to the DNA bridge in the field, with the image on the right from this set showing an expansion of the field. Scale bar, 10 µm. *Right* shows the quantification of DNA bridges relative to the total cells in the field.

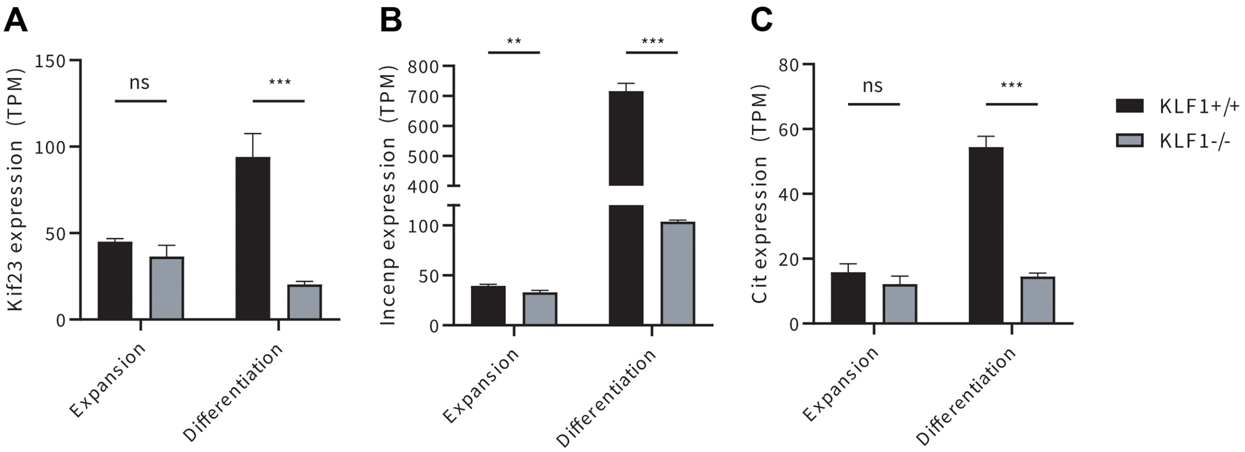

Fig. S4. Gene expression analysis in expansion/differentiating ESREs in WT and *Klf1*-/- of selected genes critical for cytokinesis. *Kif23* (A), *Incenp* (B), citron kinase (*Cit*) (C). Data is from biological triplicates analyzed for each condition. *** p<0.001; ** p<0.01; ns not significant.

Journal of Cell Science • Supplementary information

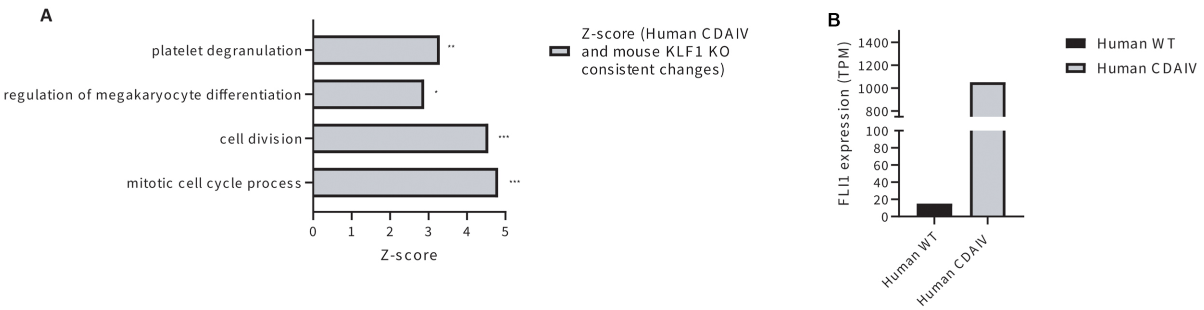

Fig. S5. Pathway analyses. (A) The z-score shows that pathways related to cell division and megakaryopoiesis are commonly dysregulated in murine *Klf1*-/- erythroid cells and in human CDA IV erythroid cells. *** p<0.001; ** p<0.01; * p<0.05; ns not significant. (B) Gene expression of FLI1, a megakaryocyte-specific gene that is normally repressed in erythroid cells, shows increased expression in erythroid cells from CDA IV patient compared to WT control, similar to that seen with murine *Klf1*-/- erythroid cells compared to WT (Fig. 2C).

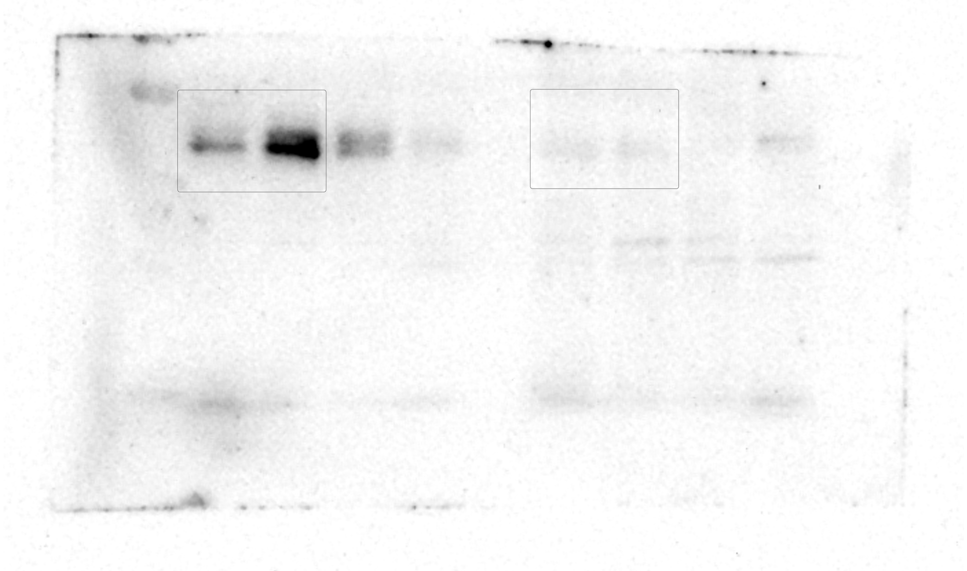

Fig. S6. Full western blot for Fig 1A; boxed regions are what was included in the figure.

Journal of Cell Science • Supplementary information

Table S1. Normalized TPM. Listed are all TPM values for the 12 samples analyzed, including triplicates of WT and *Klf1-/-* erythroid cells, each under expansion (amplifying) and differentiating conditions. Processing of the raw data was as described in Materials and Methods. These data form the basis for the graphs in Figs. 1-4, S1, S2, S4, S5.

Available for download at
https://journals.biologists.com/jcs/article-lookup/doi/10.1242/jcs.264036#supplementary-data

Table S2. Principle component analysis. x- and y-coordinate values for the data in Fig. 1B are shown.

Available for download at
https://journals.biologists.com/jcs/article-lookup/doi/10.1242/jcs.264036#supplementary-data

Journal of Cell Science • Supplementary information

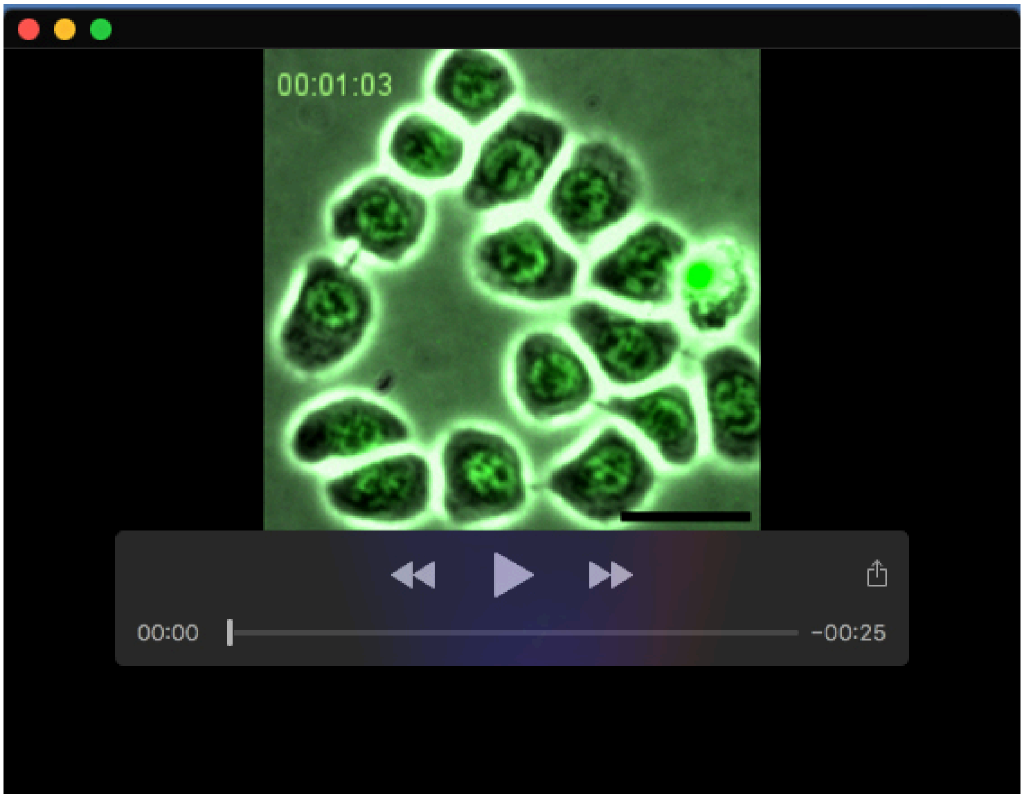

Movie 1. Time lapse of WT cells. Related to Fig. 6A.

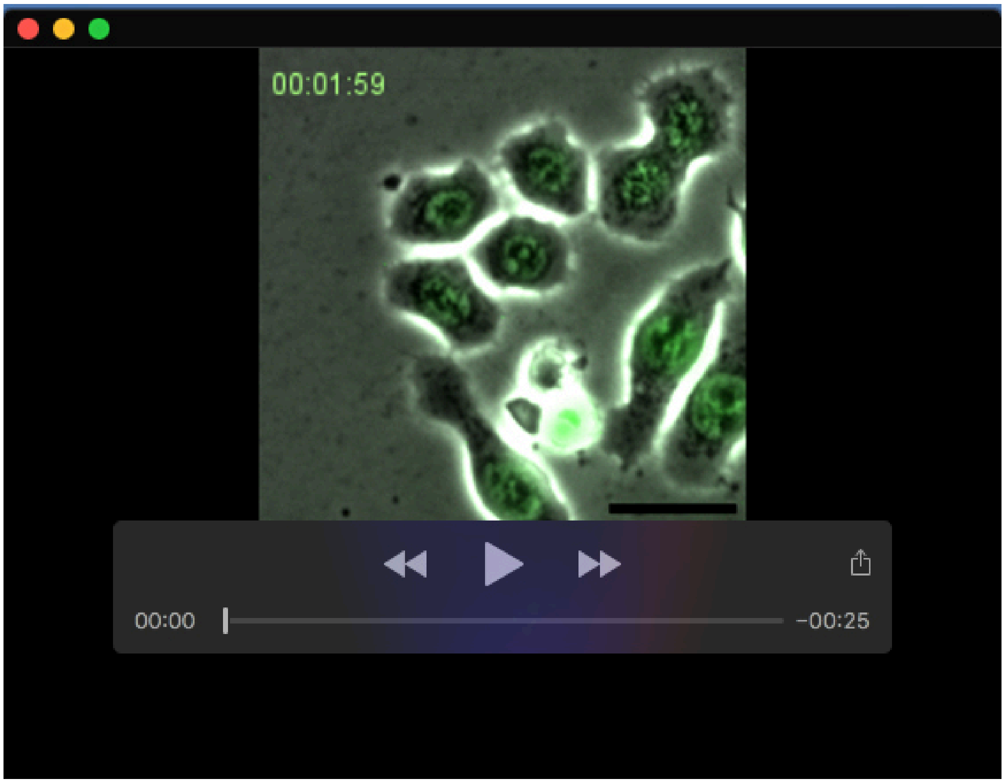

Movie 2. Time lapse of *Klf1-/-* cells. Related to Fig. 6A.

Journal of Cell Science • Supplementary information

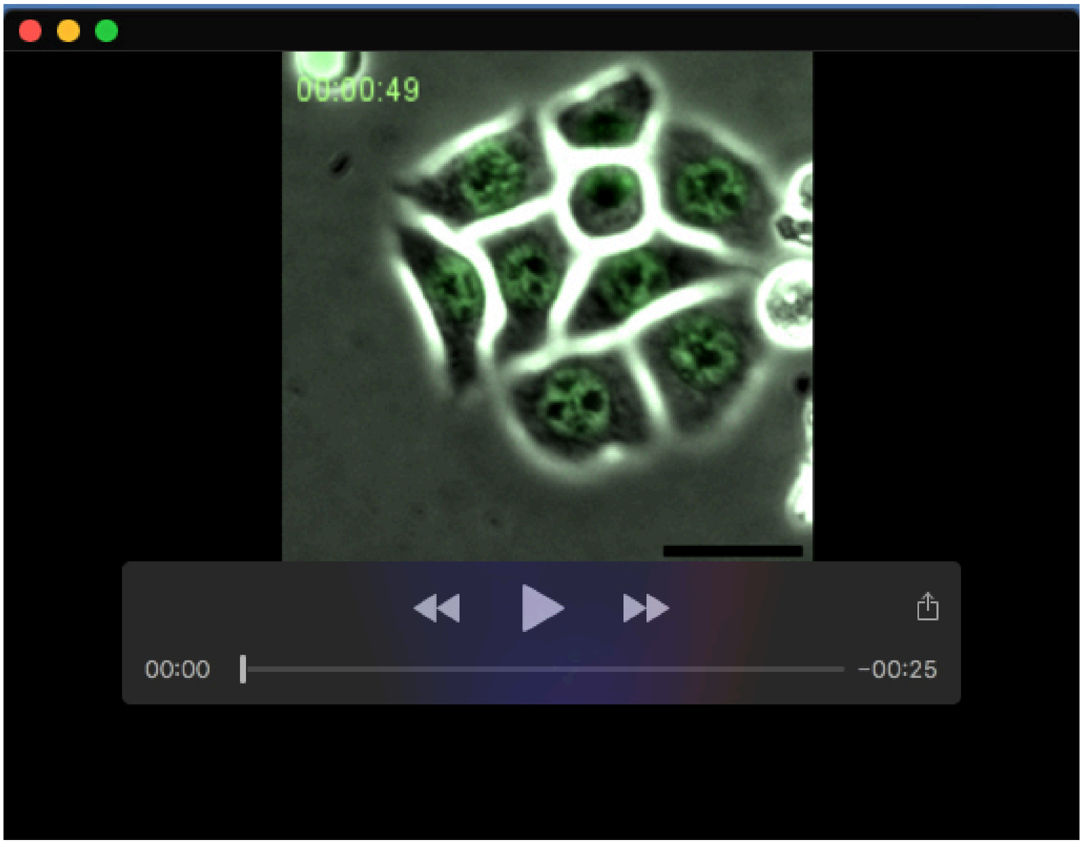

Movie 3. Additional time lapse of *Klf1-/-* cells.

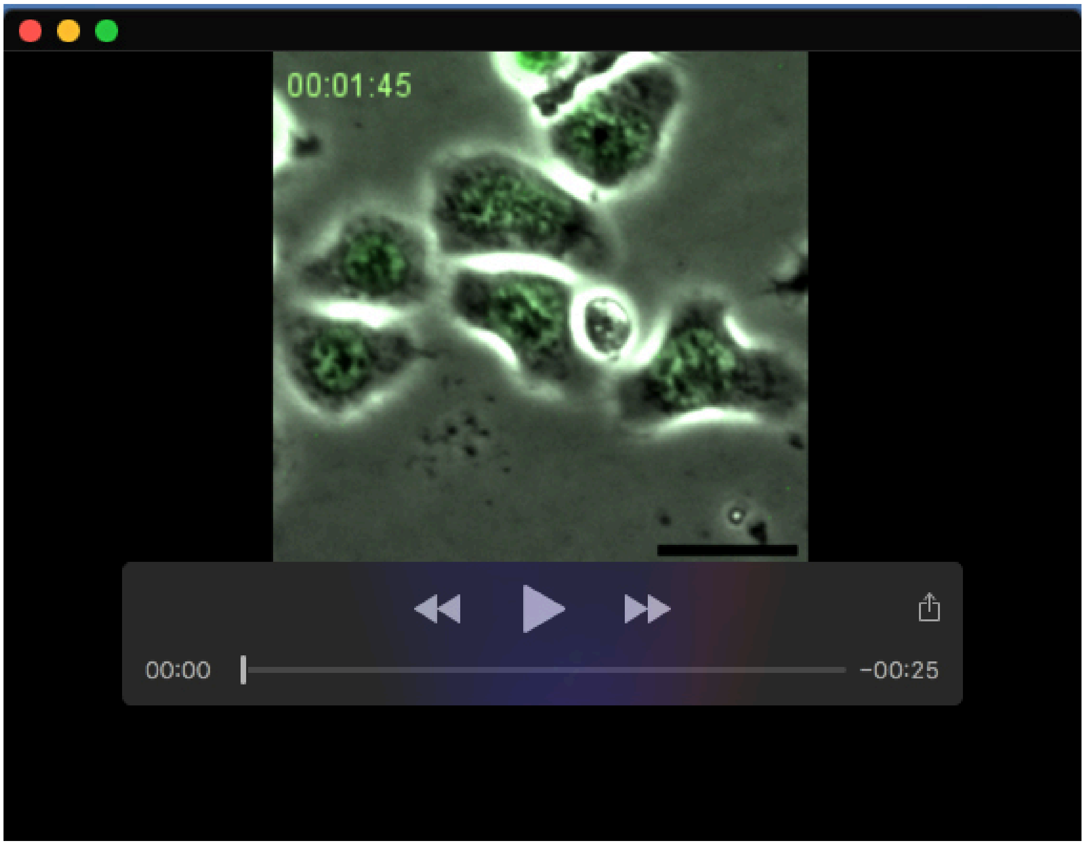

Movie 4. Additional time lapse of *Klf1-/-* cells.

Journal of Cell Science • Supplementary information
